# EMC3 regulates trafficking and pulmonary toxicity of the *SFTPC^I73T^* mutation associated with interstitial lung disease

**DOI:** 10.1172/JCI173861

**Published:** 2024-10-15

**Authors:** Xiaofang Tang, Wei Wei, Yuqing Sun, Timothy E. Weaver, Ernesto S. Nakayasu, Geremy Clair, John M. Snowball, Cheng-Lun Na, Karen S. Apsley, Emily P. Martin, Darrell N. Kotton, Konstantinos-Dionysios Alysandratos, Jiuzhou Huo, Jeffery D. Molkentin, William A. Gower, Xinhua Lin, Jeffrey A. Whitsett

**Affiliations:** 1 State Key Laboratory of Genetic Engineering, Greater Bay Area Institute of Precision Medicine (Guangzhou), School of Life Sciences, Shanghai Key Laboratory of Lung Inflammation and Injury, Zhongshan Hospital, Fudan University, Shanghai, China .; 2 Perinatal Institute, Divisions of Neonatology, Perinatal and Pulmonary Biology, Cincinnati Children’s Hospital Medical Center, Cincinnati, Ohio, USA.; 3 Biological Sciences Division, Pacific Northwest National Laboratory, Richland, Washington, USA.; 4 Department of Medicine, The Pulmonary Center, Center for Regenerative Medicine, Boston University School of Medicine, Boston, Massachusetts, USA.; 5 Division of Molecular Cardiovascular Biology, Cincinnati Children’s Hospital Medical Center, Cincinnati, Ohio, USA.; 6 Division of Pediatric Pulmonology and Program for Rare and Interstitial Lung Disease, University of North Carolina School of Medicine, Chapel Hill, North Carolina, USA.

**Keywords:** Cell biology, Pulmonology, Protein traffic

## Abstract

The most common mutation in surfactant protein C gene (*SFTPC*), *SFTPC^I73T^*, causes interstitial lung disease with few therapeutic options. We previously demonstrated that EMC3, an important component of the multiprotein endoplasmic reticulum membrane complex (EMC), is required for surfactant homeostasis in alveolar type 2 epithelial (AT2) cells at birth. In the present study, we investigated the role of EMC3 in the control of SFTPC^I73T^ metabolism and its associated alveolar dysfunction. Using a knock-in mouse model phenocopying the I73T mutation, we demonstrated that conditional deletion of *Emc3* in AT2 cells rescued alveolar remodeling/simplification defects in neonatal and adult mice. Proteomic analysis revealed that *Emc3* depletion reversed the disruption of vesicle trafficking pathways and rescued the mitochondrial dysfunction associated with I73T mutation. Affinity purification-mass spectrometry analysis identified potential EMC3 interacting proteins in lung AT2 cells, including valosin containing protein (VCP) and its interactors. Treatment of *Sftpc^I73T^* knock-in mice and *SFTPC^I73T^*-expressing iAT2 cells derived from *SFTPC^I73T^* patient-specific iPSCs with the VCP inhibitor CB5083 restored alveolar structure and SFTPC^I73T^ trafficking, respectively. Taken together, the present work identifies the EMC complex and VCP in the metabolism of the disease-associated SFTPC^I73T^ mutant, providing therapeutical targets for SFTPC^I73T^-associated interstitial lung disease.

## Abstract 2

In this manuscript, we demonstrated that inhibition of EMC3 and its interacting protein VCP altered SFTPCI73T trafficking, restored mitochondrial function in alveolar type 2 cells, and rescued alveolar defects caused by SFTPCI73T mutation.

## Introduction

Pulmonary surfactant lipids and proteins, synthesized and secreted by alveolar type 2 epithelial (AT2) cells, are required for the adaptation to air breathing during evolution (1). The gene encoding surfactant protein C, *Sftpc*, emerged in lungfish as a gene critical for the water-to-land transition (2). Surfactant proteins B and C (SP-B and SP-C) are synthesized as larger proproteins that are proteolytically processed during their transit from the endoplasmic reticulum (ER) to specialized storage organelles termed lamellar bodies (LBs). The fully processed mature SP-B and SP-C interact with surfactant lipids to form a lipid protein complex required for surfactant function and homeostasis (3).

While expression of SP-B is essential to perinatal respiration and postnatal survival (4), SP-C is dispensable for postnatal lung function but is required for homeostatic responses to lung injury (5). Monoallelic variants in the *SFTPC* gene are associated with diffuse parenchymal lung disease in children and adults (6, 7). The most common disease-associated *SFTPC* mutation, a missense isoleucine-to-threonine substitution at position 73 (I73T), causes autosomal dominantly inherited interstitial lung disease (ILD) (8, 9) with phenotypic variability ranging from mild respiratory symptoms to respiratory failure in infancy and childhood to idiopathic pulmonary fibrosis-like (IPF-like) disease in adults (10, 11).

Findings from human and mouse studies demonstrated abnormal trafficking and processing of SP-C(I73T) proprotein, causing AT2 cell toxicity, inhibition of macroautophagy, and impairment of cellular proteostasis and mitophagy (12–15). Current mouse and cell culture models expressing SP-C(I73T) model the effects of acute and high levels of expression, perhaps consistent with pathology seen in clinical exacerbations. To assess the natural history of *SFTPC^I73T^*-related disease, we inserted the I73T mutation into the mouse *Sftpc* locus. Subsequent morphological, biochemical, and proteomic analysis indicated that the knock-in mouse model phenocopied the mistrafficking and misprocessing of SP-C(I73T) and multiple aspects of AT2 cell dysfunction.

EMC3 is a key subunit of the ER membrane complex (EMC), which plays essential roles in the routing and processing of proteins through the ER (16–19). EMC3 is required for surfactant homeostasis at birth, regulating the stability of the surfactant phospholipid transporter ABCA3 and the routing of surfactant proteins SP-B and SP-C in vivo (20). In the current study, we identified the role of EMC3 in the pathogenesis of alveolar abnormalities caused by SP-C(I73T). Postnatal deletion of *Emc3* in AT2 cells altered the mistrafficking of SP-C(I73T), rescued mitochondrial function, and restored alveolar morphology. We identified valosin containing protein (VCP) as an interacting partner with EMC3. Inhibition of EMC3 or VCP restored alveolar morphology in vivo and restored homeostasis in patient-specific induced AT2 (iAT2) cells derived from *SFTPC^I73T^* patient-specific induced pluripotent stem cells (iPSCs), supporting the concept that EMC3, VCP, and other components of the EMC complex provide a framework for development of new therapies for *SFTPC^I73T^*-associated ILD and other disorders caused by similar mistrafficking of variant proteins.

## Results

### Generation of a constitutive Sftpc^I73T^ knock-in mouse model.

**Figure 1:**
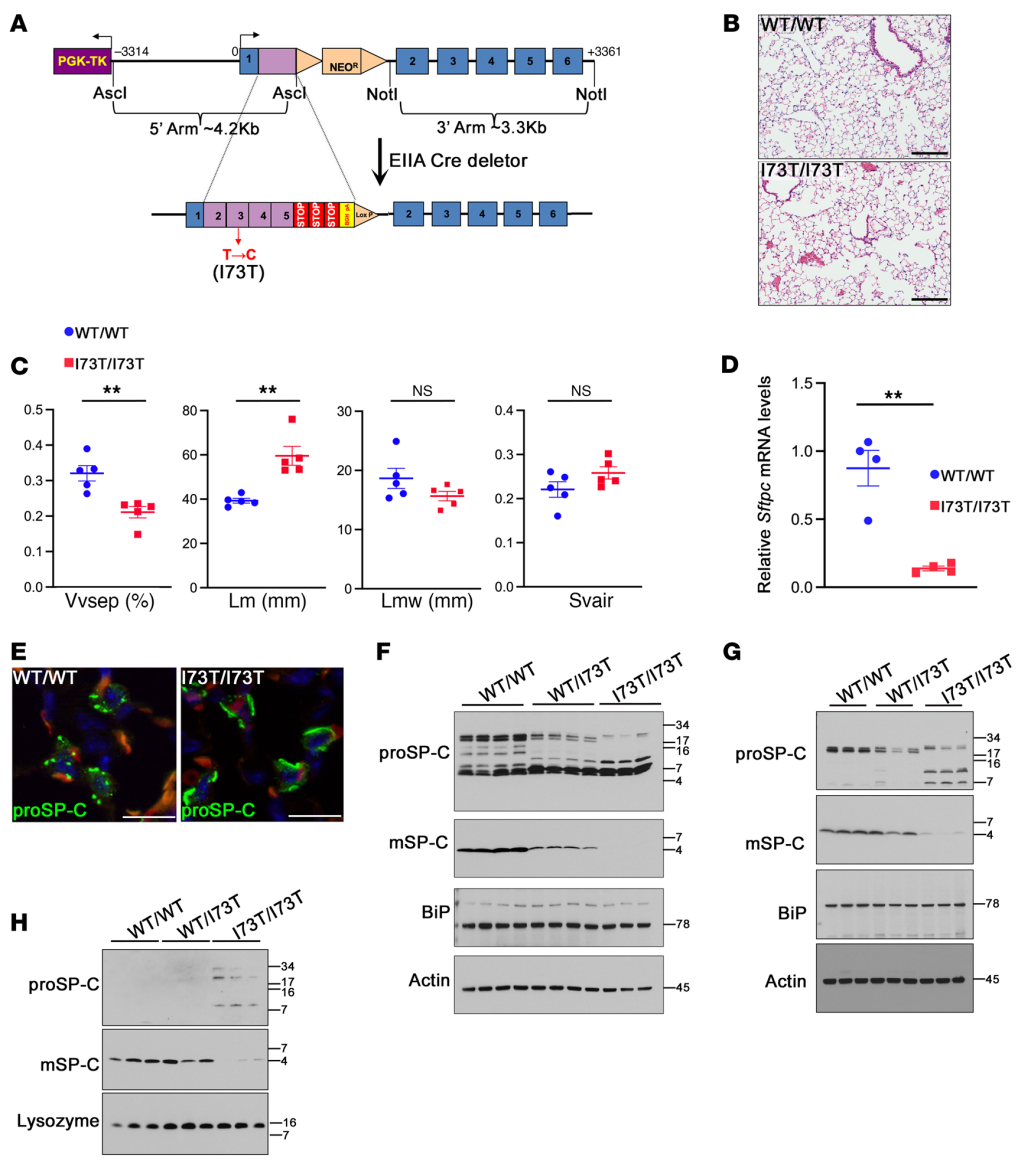
Generation of *Sftpc^I73T^* knock-in mouse model. (**A**) Design of the *Sftpc^I73T^* knock-in allele. Founder mice carrying the knock-in allele were crossed to EIIA-Cre deleter mice to excise the neomycin resistance cassette. (**B**) Representative H&E-stained sections from 6–8 week-old *WT/WT* and *I73T/I73T* mice. Scale bars: 200 μm. (**C**) Quantitative morphometry of **B** using ImageJ expressed as the volume density of alveolar septa (V_Vsep_), mean linear intercept of the airspaces (Lm), the mean transsectional wall length (Lmw), and the surface area density of the air spaces (S_Vair_). *I73T/I73T* lungs showed significantly reduced volume density of alveolar septa and increased mean linear intercept of the airspaces. Mean ± SEM; **P < 0.01 using student *t* test, *n* = 5. (**D**) Reduced *Sftpc* mRNA in isolated AT2 cells from 8-week-old *I73T/I73T* mice compared with *WT/WT* mice. Levels of the *Sftpc* transcript were normalized to that of *18S* by qPCR. Mean ± SEM; **P < 0.01 using unpaired, 2-tailed Student’s *t* test, *n* = 4/group. (**E**) 8-week-old I73T/I73T and WT/WT lung sections were stained for SP-C proprotein (proSP-C, green) and DAPI (blue). WT proSP-C is detected in a punctate pattern while proSP-C(I73T) is detected as a dense staining stripe near the cell surface. Red signal is autofluorescence of erythrocytes. Scale bars: 20 μm. (**F** and **G**) Western blot of lysates of isolated AT2 cells (**F**) or whole lung homogenates (**G**) from 6–8 week-old *WT/WT* and *I73T/I73T* mice. Processing of SP-C(I73T) proprotein into mature peptide (mSP-C) is decreased and processing intermediates are increased. (**H**) Western blot of bronchoalveolar lavage fluid (BALF) detected secreted proteins from 6–8 week-old *WT/WT* and *I73T/I73T* mice. Decreased mature SP-C and increased proSP-C(I73T) processing intermediates were present in BALF of *I73T/I73T* mice. Lysozyme was used as a loading control.

To produce a mouse model constitutively expressing SP-C(I73T), we inserted a mouse cDNA construct encoding the 218 T>C mutation into the mouse *Sftpc* locus in frame with exon 1, resulting in an amino acid substitution at position 73 (I73T) of the mouse proSP-C (Figure 1A). The floxed neomycin (neo) resistance cassette was excised by breeding mice to an Ella-Cre transgenic line, resulting in germline deletion of the neomycin cassette (Figure 1A). In all of the following experiments, we used the neomycin-free *Sftpc^I73T^* knock-in allele. As early as postnatal day 7, *Sftpc^I73T/I73T^* mice (referred to as *I73T/I73T*) had simplified alveoli (shown as focal airspace enlargement) in the peripheral lung (Figure 1, B and C and Supplemental Figure 1; supplemental material available online with this article; https://doi.org/10.1172/JCI173861DS1). Subtle airspace enlargement was observed in *Sftpc^WT/I73T^* (referred to as *WT/I73T*) lungs when compared with *Sftpc^WT/WT^* controls (referred to as *WT/WT*) (Supplemental Figure 1, A and B). No obvious fibrosis was detected in *I73T/I73T* lungs as measured by hydroxyproline assay and quantitative reverse transcription PCR (qRT-PCR) on extracellular matrix genes *Acta2*, *Col1a1*, and *Col3a1* (Supplemental Figure 1, C and D). AT2 cells from *I73T/I73T* mice expressed *Sftpc* transcripts at approximately 20% of the levels in control AT2 cells (Figure 1D). Compared with the intracellular punctate distribution of WT SP-C proprotein (proSP-C(WT)) in AT2 cells, SP-C(I73T) proprotein accumulated (Supplemental Figure 2) with dense staining detected in proximity to cell surfaces (Figure 1E). In AT2 cell lysates, whole lung homogenates, and BALF, intracellular and secreted proSP-C(I73T) was detected at higher molecular weights and was not processed efficiently into the mature SP-C peptide, with misprocessed peptides accumulating as multiple intermediates (Figure 1, F–H). BiP (Figure 1, F and G), eIF2-α phosphorylation, ATF4, CHOP, and Xbp1 splicing (Supplemental Figure 3) were detected at similar levels among different genotypes, indicating that proSP-C(I73T) was not subject to ER-associated degradation (ERAD) in vivo. Autophagy, which manifested in some patients with *SFTPC^I73T^* and a mouse model expressing acute and high levels of SP-C(I73T) (12–15), was not altered in the *I73T/I73T* AT2 cells, as reflected by unchanged protein levels of P62 and LC3B (Supplemental Figure 3A). With no alterations in autophagy and pulmonary fibrosis — likely due to low expression levels of SP-C(I73T) — findings in the *Sftpc^I73T^* knock-in mouse model are consistent with previous studies demonstrating abnormal trafficking and processing of the mutant protein, and the knock-in mouse model is suitable for study of the primary effects of SP-C(I73T) expression in vivo.

### AT2-specific deletion of Emc3 rescued alveolar simplification in Sftpc^I73T^ neonates.

**Figure 2:**
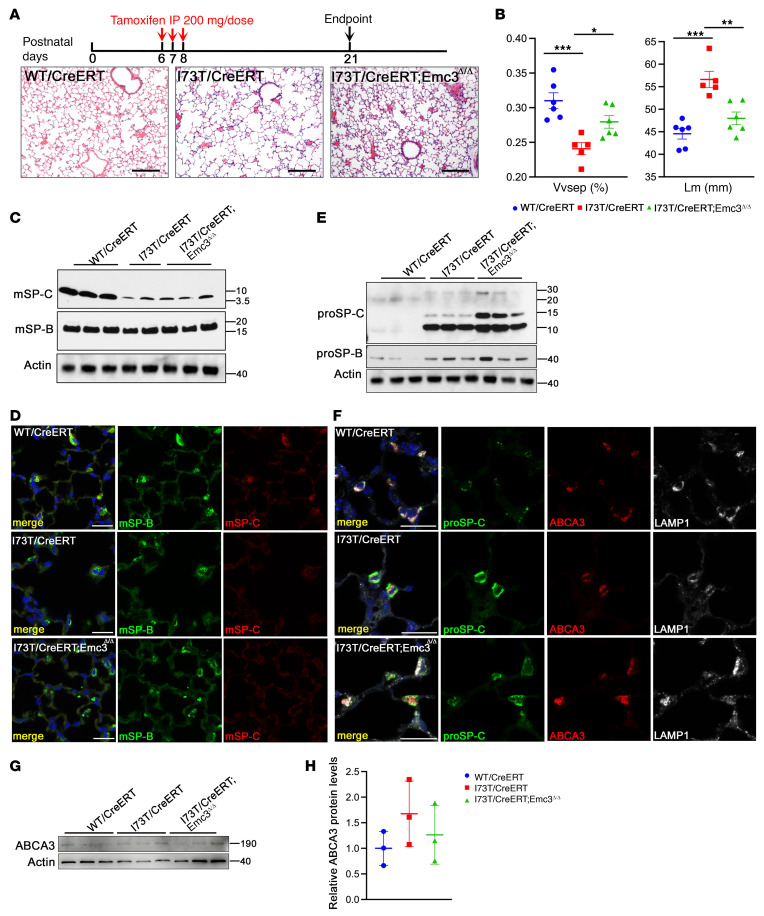
Deletion of *Emc3* rescued I73T-associated alveolar simplification in the neonatal mice. (**A**) AT2-specific deletion of *Emc3* was induced by injection of tamoxifen to neonatal mice on P6, 7 and 8. Lung tissue was analyzed on P21. Representative H&E-stained lung sections are shown. Scale bars: 200 μm. (**B**) ImageJ was used to quantify the H&E staining results in **A**. Both defects of volume density of alveolar septa (V_Vsep_) and mean linear intercept of the airspaces (Lm) in *I73T/CreERT* lungs were rescued by deletion of *Emc3*. Mean ± SEM; *P < 0.05, **P < 0.01, ***P < 0.001 using 1-way ANOVA multiple comparisons test, *n* = 6 (*WT/CreERT*), *n* = 5 (*I73T/CreERT*), *n* = 6 (*I73T/CreERT;Emc3*^Δ*/*Δ^). (**C**) Whole lung homogenates from mice treated as in **A** were prepared on P21 for Western blotting. Mature SP-B (mSP-B) levels were similar among 3 groups. Reduced levels of mSP-C were detected in *I73T/CreERT* and *I73T/CreERT; Emc3*^Δ*/*Δ^ lungs. (**D**) Lung sections were prepared from P21 mice treated as in **A** and stained for mSP-B (green), mSP-C (red) and DAPI (blue). While mSP-B was unchanged, mSP-C was decreased in *I73T/CreERT* and *I73T/CreERT; Emc3*^Δ*/*Δ^ lungs. Scale bars: 20 μm. (**E**) Western blot of AT2 cell lysates prepared from P21 mice treated as in **A**. (**F**) Lung sections were prepared from P21 mice treated as in **A** and stained for proSP-C (green), ABCA3 (red), LAMP1 (white), and DAPI (blue). Deletion of *Emc3* did not change ABCA3 staining or its colocalization with LAMP1-positive vesicles, while it decreased staining of proSP-C(I73T) near the plasma membrane and caused diffuse intracellular staining. Scale bars: 20 μm. (**G**) Western blot of AT2 cell lysates prepared from P21 mice treated as in **A**. (**H**) Quantification of Western blots in **G**. ABCA3 protein levels were normalized to that of Actin.

We previously demonstrated that loss of EMC3 in embryonic lung epithelial cells caused accumulation of proSP-C in the multivesicular bodies (MVB) and reduced its processing to the mature SP-C peptides (mSP-C) (20). Since EMC3 and other EMC subunits, including EMC4, are regulated simultaneously at protein levels (20), lacking suitable EMC3 antibody for immunostaining on lung sections, we examined the subcellular distribution of EMC4 in AT2 cells in our *Sftpc^I73T^* knock-in mouse model (Supplemental Figure 4). Distinct patterns of EMC4 staining were detected in AT2 cells from *I73T/I73T* mutant and *WT/WT* control mice, with dense staining near cell surfaces in *I73T/I73T* AT2 cells, contrasting with cytoplasmic puncta seen in controls, suggesting that EMC is involved in the regulation of SP-C(I73T) proteostasis. To delete *Emc3* in postnatal AT2 cells, we used a tamoxifen-inducible AT2-specific Cre allele, *Sftpc-CreERT*. In contrast to the indispensable role of EMC3 in perinatal surfactant metabolism (20), deletion of *Emc3* in postnatal AT2 cells did not alter lung morphology or subcellular localization of surfactant proteins SP-B and SP-C (Supplemental Figure 5). Compared with the normal lung structure in *Sftpc^WT/CreERT^* (referred to as *WT/CreERT*) mice, *Sftpc^I73T/CreERT^* (referred to as *I73T/CreERT*) mice demonstrated alveolar simplification on postnatal day 21 (P21) (Figure 2A). When *Emc3* deletion was induced in *Sftpc^I73T/CreERT^;Emc3^fl/fl^* (referred to as *I73T/CreERT;Emc3*^Δ*/*Δ^) neonatal mice, lung morphology was remarkably restored (Figure 2, A and B) on P21. Alveolar protection was not due to increased mature SP-C production, since no major changes in mSP-C(I73T) levels were detected (Figure 2, C and D). *Emc3* deletion failed to ameliorate the increased protein levels of proSP-C(I73T) or proSP-B in SP-C(I73T)–expressing AT2 cells (Figure 2E), suggesting the concept that restoration of alveolar structure was not caused by changes in SP-C(I73T) protein levels. Unlike embryonic *Emc3* deletion, ABCA3 stability was not altered by deletion of *Emc3* (Figure 2, F–H), demonstrating a dispensable role of EMC3 in maintaining postnatal ABCA3. Deletion of *Emc3* decreased the dense periplasma membrane staining of proSP-C(I73T) (Figure 2F), thus, altered transport of proSP-C(I73T) may contribute to the alveolar simplification defect associated with the mutation.

### Emc3 deletion rescued trafficking defects and mitochondrial dysfunction caused by SP-C(I73T).

**Figure 3:**
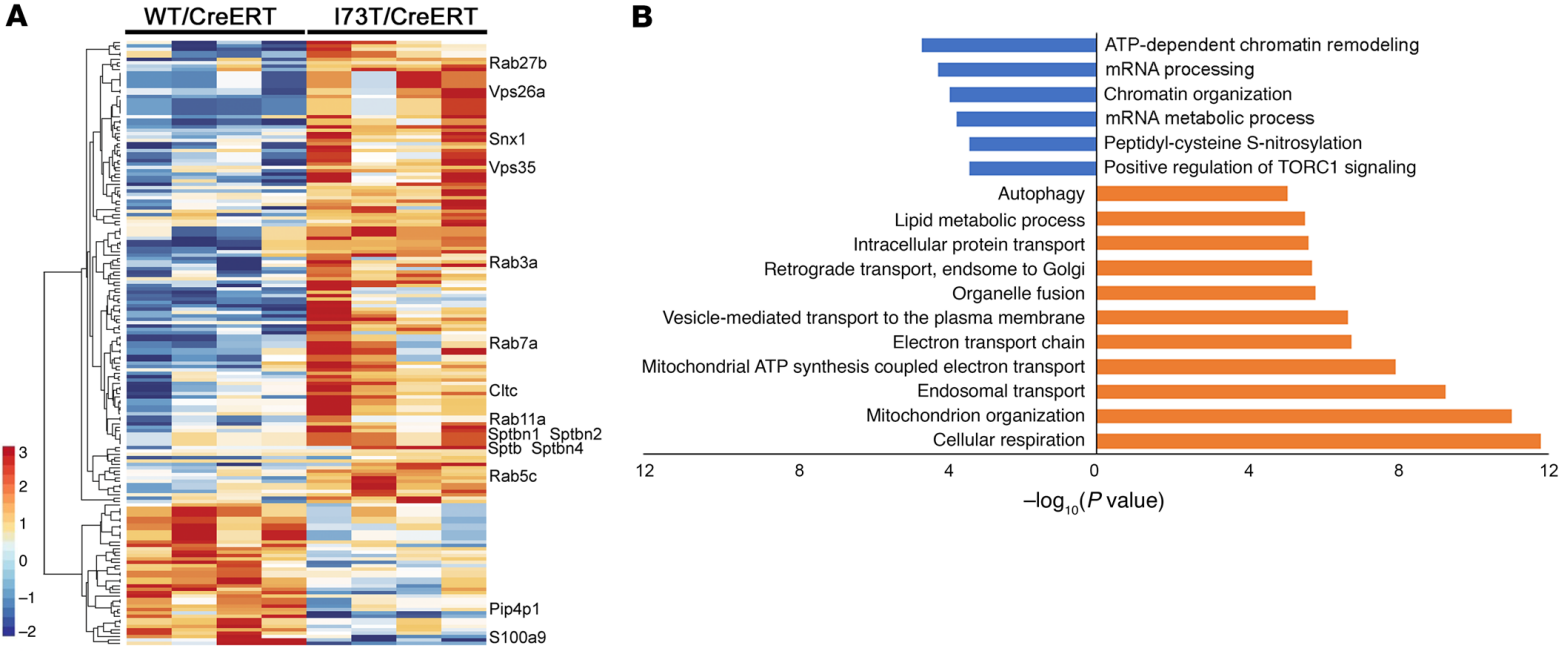
Proteomic changes caused by expression of SP-C(I73T). (**A**) Protein sequencing data were obtained from AT2 cells sorted from P21 control (*WT/CreERT*) and *Sftpc^I73T^* heterozygous (*I73T/CreERT*) mice treated with tamoxifen as in Figure 2A. Protein concentrations in AT2 cells from *I73T/CreERT* were compared with those in *WT/CreERT* mice. Proteins significantly altered were unbiasedly clustered and visualized in a *z* score–normalized heatmap. (**B**) Functional enrichment analyses of the significantly changed proteins in **A** were performed using Toppfun. A subset of significant relationships was represented by graphing the corresponding –log_10_ (*P* value). Blue represents proteins decreased and red represents those increased in AT2 cells from *I73T/CreERT* mice compared with *WT/CreERT*.

To further investigate mechanisms underlying *Sftpc^I73T^*-associated pulmonary pathology and its rescue by *Emc3* deletion, we isolated AT2 cells and compared proteomic profiles from *WT/CreERT*, *I73T/CreERT*, and *I73T/CreERT;Emc3*^Δ*/*Δ^ mice on P21. We identified 136 proteins increased and 41 decreased by expression of SP-C(I73T) (Figure 3, A and B). Increased proteins were enriched in those involved in mRNA processing and chromatin remodeling; S100A9, a calcium-binding protein promoting microtubule polymerization (21) and phagocytosis (22), and PIP4P1, a protein contributing to amino acid–induced mTORC1 activation (23), were decreased by SP-C(I73T). A number of proteins involved in vesicular trafficking (Figure 3 and Supplemental Figure 6), including cell-cortex spectrin complex (Sptb, Sptbn1/2/4), Clathrin (Cltc), Rab GTP-binding proteins (Rab3a, Rab5c, Rab7A, Rab11a, Rab27b), and retromer complex (Vps26a, Vps35, Snx1), as well as proteins involved in mitochondrial structure and function (Figure 3) were increased in *I73T/CreERT* AT2 cells. This group of proteins delineates the energy-dependent trafficking route, involving endocytosis, recycling to the cell surface, and trafficking via endolysosomes. A small group of proteins involved in autophagy were induced in AT2 cells expressing SP-C(I73T) (Figure 3). While no significant impairment in autophagic flux was detected, as measured by LC3B and P62 (Supplemental Figure 3A), this change indicated that the cellular homeostasis was under challenge to some extent, which may be exacerbated with higher doses of SP-C(I73T) or in some pathological conditions (13–15, 24).

**Figure 4:**
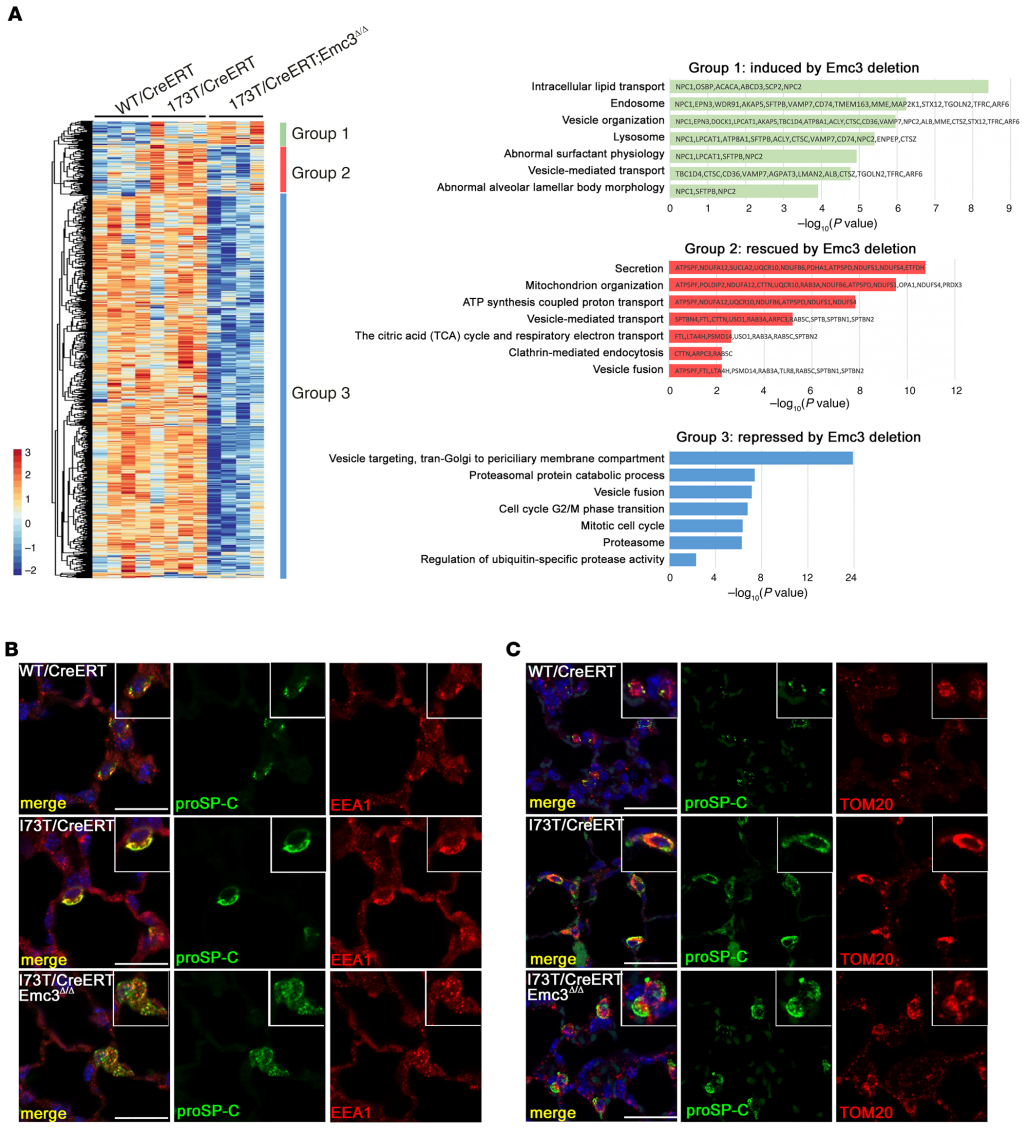
Loss of *Emc3* rescues trafficking defects and mitochondria dysfunction caused by SP-C(I73T). (**A**) Protein sequencing data were obtained from AT2 cells sorted from control (*WT/CreERT*), *Sftpc^I73T^* heterozygous (*I73T/CreERT*), and *I73T/CreERT;Emc3*^Δ*/*Δ^ mice on P21 after treatment with tamoxifen as in Figure 2A. Protein levels in *I73T/CreERT;Emc3*^Δ*/*Δ^ AT2 cells were compared with that in the other 2 genotypes and proteins significantly altered in 1 or more conditions were unbiasedly clustered and visualized in a *z* score–normalized heatmap. Group 1, proteins induced in AT2 cells from *I73T/CreERT;Emc3*^Δ*/*Δ^ lungs; Group 2, proteins rescued by *Emc3* deletion (induced in *I73T/CreERT* and reversed in *I73T/CreERT;Emc3*^Δ*/*Δ^); Group 3, proteins only repressed in *I73T/CreERT;Emc3*^Δ*/*Δ^ AT2 cells. Functional enrichment analyses of these proteins were performed using Toppfun. A subset of significant relationships was represented by graphing the corresponding –log_10_ (*P* value). (**B**) Lung sections were prepared from P21 mice treated with tamoxifen as in Figure 2A and stained for proSP-C (green), an early endosome marker, EEA1 (red), and DAPI (blue). Diffuse staining of EEA1 in AT2 cells is shown in *WT/CreERT* controls. SP-C(I73T) accumulated with EEA1 in close proximity to plasma membranes. *Emc3* deletion restored the diffuse intracellular distribution of both proSP-C(I73T) and EEA1. (**C**) Lung sections were prepared from P21 mice treated with tamoxifen as in Figure 2A and were stained for proSP-C (green), TOM20 (red), and DAPI (blue). Mitochondrial outer membrane protein TOM20 accumulated in SP-C(I73T)–expressing AT2 cells and was restored by deletion of *Emc3*. Scale bars: 20μm.

The effects of *Emc3* deletion on AT2 cell proteins were assessed in *I73T/CreERT;Emc3*^Δ*/*Δ^ mice, identifying those increased and decreased compared with AT2 cells from *WT/CreERT* (59 and 916 respectively) and *I73T/CreERT* (16 and 960 respectively). Proteins induced in *Sftpc^I73T^* AT2 cells by *Emc3* deletion (Figure 4A, Group 1) included proteins involved in surfactant physiology, lysosome function, and lamellar body morphology, perhaps consistent with enlarged lamellar bodies in AT2 cells from *I73T/CreERT;Emc3*^Δ*/*Δ^ mice (Figure 2F and Supplemental Figure 7). Proteins repressed in *Sftpc^I73T^* AT2 cells by *Emc3* deletion (Figure 4A, Group 3 and Supplemental Table 1) included proteins involved in vesicular trafficking, cell cycle regulation, and proteasomal catabolic processes, consistent with previously reported roles for EMC3 in ERAD (25).The group of proteins induced by the *Sftpc^I73T^* mutation and restored by deletion of *Emc3* (Figure 4A, Group 2), consisted of proteins involved in mitochondrial function and vesicular transport (Figure 4A). Immunostaining of *I73T/CreERT; Emc3*^Δ*/*Δ^ AT2 cells with endosomal markers EEA1 and Rab11 demonstrated their diffuse intracellular staining patterns characteristic of normal AT2 cells (Figure 4B and Supplemental Figure 6). Staining for the mitochondrial outer membrane protein TOM20 and electron microscopic analysis on AT2 cells revealed that *Emc3* deletion restored the abnormal accumulation and distribution of mitochondria associated with SP-C(I73T) expression (Figure 4C and Supplemental Figure 7). Notably, the 2 proteins decreased by *Sftpc^I73T^* mutation, S100A9 and PIP4P1, were normalized by the deletion of *Emc3*.

### AT2-specific deletion of Emc3 rescued alveolar simplification and mitochondrial dysfunction in adult Sftpc^I73T^ mice.

**Figure 5:**
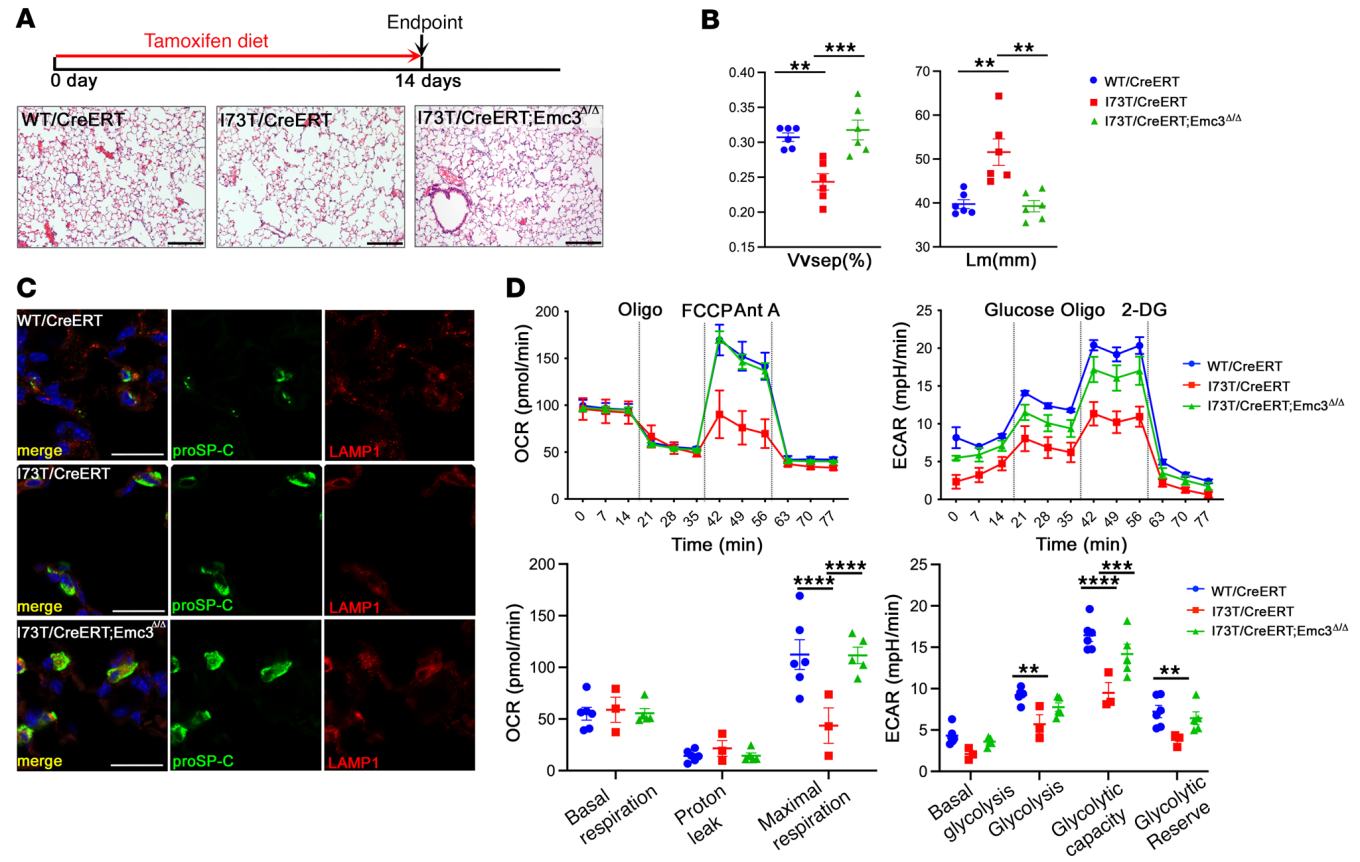
Loss of *Emc3* rescues I73T-associated alveolar simplification and mitochondrial dysfunction in adult mice. (**A**) AT2-specific *Emc3* deletion was induced by administration of tamoxifen chow to control (*WT/CreERT*), *Sftpc^I73T^* heterozygous (*I73T/CreERT*), and *I73T/CreERT;Emc3*^Δ*/*Δ^ adult mice (6–8 weeks of age). Lung tissues were obtained 14 days later. Representative H&E-stained lung tissue is shown. Scale bars: 200 μm. (**B**) ImageJ was used to quantify the H&E staining results in **A**. Both defects of volume density of alveolar septa (V_Vsep_) and mean linear intercept of the airspaces (Lm) in *I73T/CreERT* lungs were rescued by deletion of *Emc3*. Mean ± SEM; **P < 0.01, ***P < 0.001 using 1-way ANOVA multiple comparisons test, *n* = 6. (**C**) Lung sections from adult mice treated as in **A** were stained for proSP-C (green), LAMP1 (red) and DAPI (blue). *Emc3* deletion restored the intracellular distribution of SP-C(I73T). Scale bar: 20 μm. (**D**) Oxygen consumption rate (OCR) and extracellular acidification rates (ECAR) of AT2 cells isolated from adult mice treated as in **A** were tested with the Seahorse XF96 analyzer using 1.2 × 10^5^ cells for each sample. OCR was measured under basal conditions, followed by addition of oligomycin (Oligo; 2 μM), FCCP (2 μM), and antimycin A (Ant A; 2.5 μM), as indicated. ECAR was measured under basal conditions followed by addition of glucose (10 nM), oligomycin (Oligo; 2 μM), and 2-DG (50 mM), as indicated. *Emc3* deletion restored maximal respiration and glycolytic capacity of AT2 cells expressing SP-C(I73T). Mean ± SEM; **P < 0.01, ****P < 0.0001 using 2-way ANOVA multiple comparisons test, *n* = 6 (*WT/CreERT*), *n* = 3 (*I73T/CreERT*), *n* = 5 (*I73T/CreERT;Emc3*^Δ*/*Δ^).

To test whether EMC inhibition restored alveolar defects in adult *Sftpc^I73T^* mice, we induced AT2-specific deletion of *Emc3* by administration of a tamoxifen diet to adult *WT/CreERT*, *I73T/CreERT,* and *I73T/CreERT;Emc3*^Δ*/*Δ^ mice (6–8 weeks of age) (Figure 5A). After 2 weeks, deletion of *Emc3* attenuated SP-C(I73T)–associated alveolar simplification (Figure 5, A and B) and restored the diffuse intracellular distribution of proSP-C(I73T) (Figure 5C). To investigate whether deletion of *Emc3* restored mitochondrial functions, we measured oxygen consumption rates (OCR) and extracellular acidification rates (ECAR) in sorted AT2 cells from tamoxifen-treated adult mice (Figure 5D). While no significant differences in basal respiration were detected by OCR, maximal respiration capacity was decreased in *I73T/CreERT* AT2 cells and restored by *Emc3* deletion. While significant differences in basal glycolysis were not observed among 3 genotypes as measured by ECAR, glycolysis, glycolytic capacity, and glycolytic reserve were reduced in *I73T/CreERT* AT2 cells, and deletion of *Emc3* significantly improved glycolytic capacity (Figure 5D). Taken together, mistrafficking of SP-C(I73T) causes mitochondrial dysfunction that may contribute to the alveolar defects in *Sftpc^I73T^* mice; *Emc3* deletion in AT2 cells was sufficient to reverse alveolar simplification caused by *Sftpc^I73T^* in both neonatal and adult mice.

### EMC3 interacts with VCP to regulate SP-C(I73T) trafficking and alveolar morphology.

**Figure 6:**
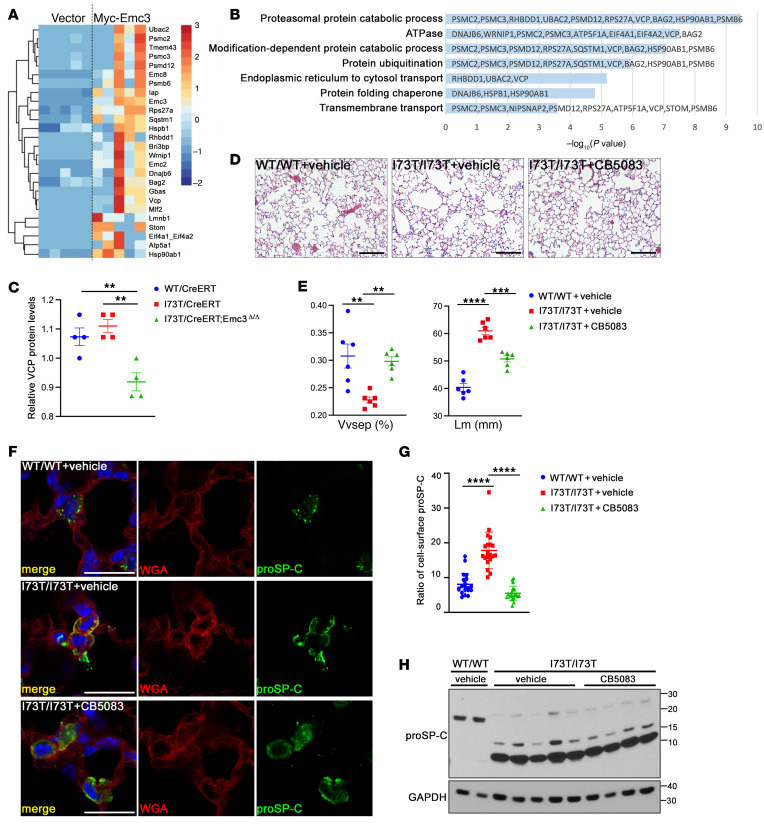
EMC3 interacts with VCP to influence SP-C(I73T) trafficking. (**A**) Mass spectrometry of proteins isolated from EMC3 coimmunoprecipitates in MLE-15 cells identified 26 proteins as potential EMC3 binding partners. For each experimental pair, MLE-15 cells were transfected with empty vector or vector encoding Myc-tagged EMC3 (Myc-EMC3). Myc antibody coimmunoprecipitates were isolated from cell lysates using μMACS c-myc Isolation Kit (Miltenyi Biotec) and analyzed by mass spectrometry. Five independent pairs of co-IP assays were performed and analyzed. The R package *apmsWAPP,* sub package TSPM, was used to determine significant EMC3 interacting partners, *P* < 0.05. Significant EMC3 PPI partners were visualized in a *z* score–transformed heatmap of the normalized spectral counts. (**B**) Functional enrichment analyses of potential EMC3 interaction partners were performed using Toppfun. A subset of significant relationships was represented by graphing the corresponding –log_10_ (*P* value). (**C**) Normalized VCP protein levels were measured from the proteomic analysis in Figure 4A. Mean ± SEM; **P < 0.01 using 2-way ANOVA multiple comparisons test, *n* = 4/group. (**D**) VCP inhibitor, CB5083, was given every other day (q.o.d.) by oral gavage at 50 mg/kg/dose to 6–8 week-old I73T/I73T mice for 14 days. Representative H&E-stained lung sections are shown. Scale bar: 200 μm. (**E**) ImageJ was used to quantify the H&E staining results in **D**. Mean ± SEM; **P < 0.01, ***P < 0.001, ****P < 0.0001 using 1-way ANOVA multiple comparisons test, *n* = 6. (**F**) Lung sections from adult mice treated in **D** were stained for proSP-C and cell-surface marker WGA. Scale bars: 20 μm. (**G**) Quantification of the ratio of cell-surface proSP-C stained in **F** by ImageJ. For each group, 20 AT2 cells from 4 mice were quantified. (**H**) Western blot of lysates from AT2 cells isolated from CB5083 or vehicle treated adult mice as in **D**. *Emc3* deletion did not change levels of proSP-C(I73T).

To identify potential EMC3 interactions in lung epithelial cells, we performed affinity-purification mass spectrometry analysis using MLE15 cells (a mouse lung epithelial cell line transformed with SV40 large T-antigen) (26) expressing Myc-tagged EMC3 to isolate EMC3 interacting proteins. A total of 26 proteins were identified in EMC3 coprecipitates (Figure 6A). Known EMC subunits, EMC2 and EMC8, were coprecipitated, as expected. Potential EMC3-interacting proteins represented several cellular processes (Figure 6B), including proteasomal protein catabolism, consistent with their loss in *Emc3*-deficient AT2 cells (Figure 4A). Similar to our previous findings in NIH3T3 cells (27), the conserved AAA ATPase VCP (also known as p97/Cdc48) (28–30) was identified as one EMC3-interacting protein in lung epithelial cells. Through its cooperation with a diversity of cofactors, VCP plays integral roles in many cellular processes, including proteostasis and vesicular trafficking. In support of a potential role of VCP in the control of SP-C(I73T) by EMC3, VCP protein was significantly reduced by *Emc3* deletion in AT2 cells of *I73T/CreERT;Emc3*^Δ*/*Δ^ mice (Figure 6C).

To further test the involvement of VCP in the control of SP-C(I73T) proteostasis, we first examined the effects of VCP inhibition in MLE-15 and human epithelial cell line HEK293T. siRNA-mediated knockdown of VCP did not change the trafficking or processing of WT SP-C protein, but rendered the cell-surface SP-C(I73T) to intracellular vesicles (Supplemental Figure 8). Next, we analyzed the in vivo role of VCP in SP-C(I73T) proteostasis by inhibiting VCP activity in adult *Sftpc^I73T^* mice. On alternate day (q.o.d.), 14 days of treatment with the VCP inhibitor, CB5083 (31), significantly improved alveolar morphology in *I73T/I73T* adult mice without causing overt toxicity (Figure 6, D and E). VCP inhibition altered proSP-C(I73T) staining, similar to that observed after *Emc3* deletion (Figure 6, F and G). The rescuing effect of CB5083 was not associated with any change in the levels of misprocessed proSP-C(I73T) intermediates (Figure 6H).

### Rescue of human SFTPC^I73T^ by inhibition of EMC3 or VCP.

**Figure 7:**
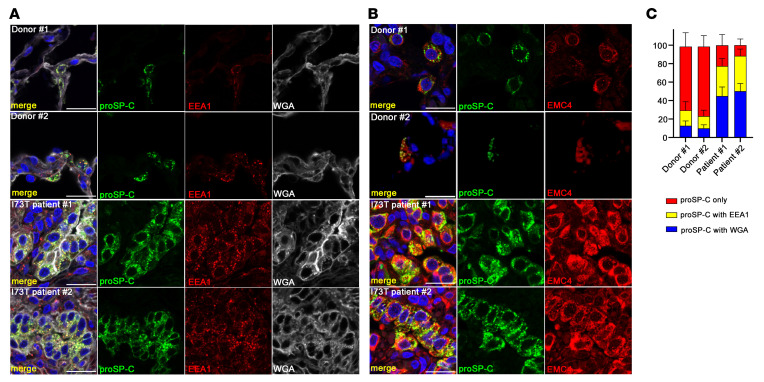
Accumulation and localization of SP-C(I73T) and EMC4 in patients with *SFTPC^I73T^*-related ILDs. Representative confocal immunofluorescence microscopy of sections of explanted lungs from normal donors and patients with *SFTPC^I73T^*-associated ILD. Scale bars: 20 μm. (**A**) Lung sections were stained for proSP-C (green), EEA1(red), WGA (white), and DAPI (blue). WGA staining was used to mark the plasma membrane. Accumulation and colocalization of proSP-C(I73T) and EEA1 was detected in lung tissues from the patients with *SFTPC^I73T^*. (**B**) Lung sections were stained for proSP-C (green), EMC4 (red), and DAPI (blue). Accumulation and distinct distribution of EMC4 was detected in AT2 cells of patients with *SFTPC^I73T^*. (**C**) Quantification of the ratio of subcellular proSP-C stained in **A** by ImageJ. Fifteen AT2 cells were quantified for each individual. Note that AT2 cells in the 2 patients accumulate more proSP-C(I73T) on the cell surface and in EEA1-positive endosomes.

Given the improvement on alveolar structure by loss or inhibition of EMC3 and VCP, we tested whether inhibition of EMC3 or VCP influenced the processing and toxicity of human SP-C(I73T) in patients with ILD. We performed proSP-C and EMC4 immunostaining in lungs from 2 patients with ILD caused by *SFTPC^I73T^*. SP-C(I73T) proproteins accumulated in AT2 cells in both individuals, colocalizing with the endosomal marker EEA1 or cell surface marker WGA, and EMC4 staining was increased, consistent with findings in *Sftpc^I73T^* knock-in mouse model (Figure 7). Likewise, *SFTPC^I73T^* AT2 cells demonstrated distinct cell surface staining pattern for VCP compared with homogenous intracellular distribution in control AT2s (Supplemental Figure 9).

**Figure 8:**
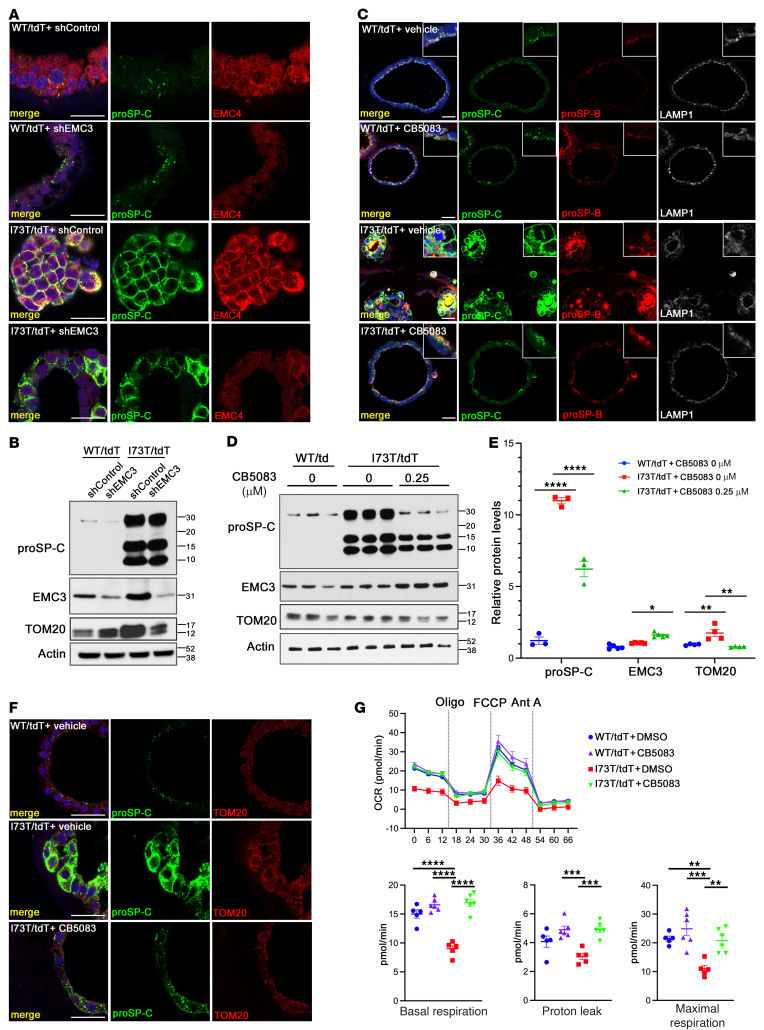
Rescue of I73T-iAT2 cells by *EMC3* shRNA and VCP inhibition. (**A**) I73T/tdT and WT/tdT iAT2 cells were transfected with control shRNA (shControl) or *EMC3* shRNA (shEMC3). Fourteen days after transfection, cells were fixed and paraffin sections were stained for proSP-C (green), EMC4 (red), and DAPI (blue). EMC4 staining was used to identify sites of *Emc3* inhibition. Scale bars: 20 μm. (**B**) Western blots were performed on lysates of iAT2 cells in **A**. In I73T/tdT iAT2 cells, shEMC3 transfection reduced EMC3 and TOM20 expression. (**C**) WT/tdT and I73T/tdT iAT2 cells were treated with DMSO (vehicle) or 0.25 μM CB5083 for 14 days and stained for proSP-C (green), proSP-B (red), LAMP1 (white), and DAPI (blue). Scale bars: 20 μm. (**D**) Western blots on lysates of iAT2 cells treated with DMSO or CB5083 as in **C**. (**E**) Quantification of Western blots in **D**. Total proSP-C (*n* = 3), EMC3 (*n* = 5), and TOM20 (*n* = 4) were normalized to that of actin. Mean ± SEM; *P < 0.05, **P < 0.01, ****P < 0.0001 using 2way ANOVA multiple comparisons test. (**F**) *I73T/tdT* iAT2 cells were treated with CB5083 as in **C** and stained for proSP-C (green), TOM20 (red), and DAPI (blue). Scale bars: 20 μm. (**G**) Oxygen consumption rate (OCR) of *WT/tdT* or *I73T/tdT* iAT2 cells treated with vehicle (DMSO) or CB5083 were tested with the Seahorse XF96 analyzer using 2 × 10^4^ cells for each sample. OCR was measured under basal conditions, followed by addition of oligomycin (Oligo; 2 μM), FCCP (2 μM), and antimycin A (Ant A; 2.5 μM), as indicated. *Emc3* deletion restored basal respiration, proton leak, and maximal respiration of *I73T/tdT* iAT2 cells. Mean ± SEM; **P < 0.01, ***P < 0.001, ****P < 0.0001 using 1-way ANOVA multiple comparisons test, *n* = 5 (WT/tdT+DMSO), *n* = 6 (WT/tdT+CB5083), *n* = 5 (I73T/tdT+DMSO), *n* = 6 (I73T/tdT+CB5083).

*SFTPC^I73T^* patient-specific iPSCs and their gene-corrected counterparts were previously engineered with a tdTomato reporter inserted into the endogenous *SFTPC* locus (14) (referred to as *I73T/tdT* and *WT/tdT,* respectively) and differentiated to iAT2 cells. proSP-C(I73T) accumulated and was misprocessed by iAT2 cells, consistent with recent findings (14). EMC4 staining was increased in *I73T/tdT* iAT2 cells (Supplemental Figure 10), consistent with findings in the *Sftpc^I73T^* mice. shRNA-mediated inhibition of *EMC3* in the iAT2 cells redistributed proSP-C(I73T) staining (Figure 8A) and did not alter proprotein levels, as assessed by Western blots (Figure 8B). Increased mitochondrial staining of TOM20 in *I73T/tdT* iAT2 was suppressed by *EMC3* inhibition (Figure 8B). Treatment of the *I73T/tdT* iAT2 cells with either *shEMC3* (Figure 8A) or the VCP inhibitor CB5083 (Figure 8C and Supplemental Figure 8) rescued alveolosphere morphology, a previously reported morphometric readout of *SFTPC^I73T^*-induced disease activity that is associated with perturbed iAT2 apical-basal polarity in this model (14). Ball-like structures formed by *I73T/tdT* iAT2 cells were restored to the monolayered alveolosphere morphology characteristic of *WT/tdT* iAT2 cells (Figure 8, A and C). In *I73T/tdT* iAT2 cells treated with CB5083, proSP-C(I73T) misprocessing was reduced (Figure 8, D and E) and its mistrafficking was inhibited, indicated by punctuate staining pattern of proSP-C(73T) and correction of the abnormal staining of endosomal markers EEA1and Rab11(Supplemental Figure 11). CB5083 treatment restored the distribution and protein levels of the mitochondrial marker TOM20 as well as mitochondrial function (Figure 8, D–G). CB5083 slightly increased EMC3 levels in *I73T/tdT* iAT2 cells (Figure 8, D and E), perhaps indicating that VCP acted downstream of EMC3 in the regulation of *SFTPC^I73T^,* and reduction of VCP activity caused a compensatory increase in EMC3 levels. Inhibition of EMC3 or VCP did not alter normal SP-C processing or alveolosphere morphology of *WT/tdT* iAT2 cells, suggesting a role of EMC3/VCP specific to the metabolism of SP-C(I73T) mutant protein.

## Discussion

We developed in vivo and in vitro models to investigate the role of EMC3 in the pathogenesis of alveolar epithelial dysfunction caused by a mutation in the surfactant protein C gene, *SFTPC^I73T^*. We developed an *Sftpc^I73T^* knock-in mouse model and used it with *SFTPC^I73T^* patient-specific iPS-derived iAT2 cells to investigate the epithelial intrinsic factors underlying lung disease caused by the mutation. We identified a role for EMC3 in the regulation of intracellular trafficking of the mutant protein. Amelioration of both the alveolar simplification and mitochondrial dysfunction associated with *SFTPC^I73T^* by deletion or inhibition of EMC3 or VCP identifies these pathways for development of pharmacological therapy of *SFTPC^I73T^*-associated lung disease. Targeting the EMC complex and VCP offers potential therapeutic approaches for other disorders caused by mistrafficking of variant proteins.

While it is widely recognized that proSP-C(I73T) undergoes mistrafficking, misprocessing, and intracellular accumulation, the mechanism by which *SFTPC^I73T^* mutation contributes to AT2 abnormalities including mitochondrial dysfunction, impaired autophagy/mitophagy/lysophagy, and, further, to severe respiratory symptoms such as pulmonary fibrosis and inflammation, is still unclear. In this study, we generated a knock-in mouse model that phenocopied the mistrafficking, misprocessing, and intracellular accumulation of SP-C(I73T) to a modest level. Histological analysis of the lung identified mild respiratory symptoms without obvious fibrosis or inflammation and, following proteomic analysis of the AT2 cells, identified primary changes as disruption in vesicular transport and mitochondrial function without markedly altered autophagy/mitophagy. Moreover, when the cell-surface accumulation of proSP-C(I73T) is rescued by inhibition of EMC3/VCP, the lung morphology as well as mitochondrial function are also restored even without a change in proSP-C(I73T) processing or total protein levels. Therefore, we hypothesize that the toxic gain-of-function associated with *SFTPC^I73T^* mutation results from mistrafficking of the SP-C(I73T) proprotein. Other factors with impact on the proteotoxic stress to AT2 cells may influence penetrance and symptom severity of *SFTPC^I73T^*-associated ILD, such as expression levels of the cytotoxic SP-C(I73T), age-related decline in mitochondrial function, mutations in mitochondrial DNA, and disruption of autophagy (32).

The present study identifies a role for EMC3 and its interacting partner VCP in the regulation of SP-C(I73T) trafficking via a mechanism distinct from that underlying its regulation of WT SP-C. We demonstrated previously that EMC3 was critical for the processing and trafficking of WT SP-C at birth via regulation of the unfolded protein response (UPR) and biogenesis of certain transmembrane proteins including cathepsin enzymes required for SP-C processing and trafficking (20). In the postnatal lungs, our current work indicated that EMC3 was dispensable for the metabolism of SP-C and ABCA3 as well as lung function in WT mice. By contrast, deletion of *Emc3* rescued AT2 cell dysfunction and alveolar abnormalities in both neonatal and adult *Sftpc^I73T^* mice, as indicated by protection from alveolar simplification. siRNA-mediated knockdown of VCP and treatment of the *Sftpc^I73T^* mice or patient-specific iAT2s with a VCP inhibitor phenocopied the ameliorating effects of *Emc3* deletion. While total protein levels and processing of proSP-C(I73T) were not consistently altered, perhaps related to the nature and proteostasis status of the cells, our work supports the concept that altering SP-C(I73T) trafficking route by EMC3/VCP inhibition is sufficient to minimize damage to AT2 cells and thus to ameliorate ILD.

How does VCP/EMC3 regulate the mistrafficking of SP-C(I73T)? Mechanisms by which *Emc3* deletion restores trafficking of SP-C(I73T) and mitochondrial function are likely to be complex, and it is presently unclear whether the mechanisms involve improved clearance/altered trafficking of proteins to or from the cell surfaces and endosomes or disruption of ER processing and folding earlier in proSP-C(I73T) biosynthesis. While VCP is a calcium-associated ATPase that plays diverse roles in protein transport, ERAD, autophagy, and cell cycle by extracting and refolding ubiquitinated proteins (33), it mediates intramembrane protein transport functions within the endocytic pathway with intracellular distribution overlapping with the EMC complex (reviewed in ref. 34). Since the I73T mutation disrupts SP-C oligo ubiquitination, EMC3 and VCP may influence SP-C(I73T) trafficking indirectly through modulating interactions with various components of the ER and endocytic machinery. Several factors, including EEA1 (35), clathrin (36), and CAV1 (37) interact with VCP to regulate their ubiquitination. During normal processing of proSP-C, the protein is targeted to MVB for final processing to the active peptide through K63-linked oligo ubiquitination (38). In contrast, SP-C(I73T) is recycled to and retained at the cell surface and is not fully processed into mature peptide (39). Given the enlarged sizes of lamellar bodies and increased levels of lysosomal proteins caused by the deletion of *Emc3*, the retained SP-C(I73T) proprotein may be subject to other proteolysis or storage sites after EMC3/VCP inhibition.

Currently, there are no effective therapies for *SFTPC^I73T^*-related ILD other than lung transplantation. Inhibition of EMC3/EMC or VCP in *Sftpc^I73T^* transgenic mice and in *SFTPC^I73T^* iAT2 cells improved mitochondrial dysfunction in association with changes in protein transport without affecting normal AT2 cell function in neonatal or adult mice, supporting potential targeting of EMC3 and VCP pathways for development of treatments to prevent or treat *SFTPC^I73T^*-related ILD. While we are not aware of EMC inhibitors at present, inhibitors of the AAA-ATPase VCP CB5083 and a second-generation molecule CB5339, have been developed and are being evaluated in clinical trials for anticancer activity. Since VCP inhibitors were designed to enhance proteotoxic stress in tumor cells and are likely to have diverse effects because of the many intracellular functions of VCP, optimization of VCP inhibitors or development of drugs to safely target either the EMC3 or VCP pathways and/or their targeted delivery to AT2 cells may make their clinical translation into ILD treatment possible. Present findings demonstrating the amelioration of AT2 cell toxicity caused by the SP-C variant protein support further study of manipulating EMC or VCP for other disorders caused by misfolding of disease-causing protein variants.

## Methods

### Sex as a biological variable.

Both male and female mice were used in this study.

### Mice.

All mice used in this study were on C57BL/6J background. The *Sftpc^I73T^* knock-in allele bearing 218 T>C point mutation was generated using the same strategy as previously described (40). The *Sftpc-CreERT* line (41) was a gift from Harold A. Chapman (University of California San Francisco, California, USA). The *Emc3^fl^* allele was generated in our laboratory as previously described (20). To activate Cre recombination, young pups were administered 200 μg of tamoxifen (Sigma-Aldrich) via i.p. injection once a day on P6, 7, and 8; 6–8 week-old adult mice were administered a tamoxifen diet (400 mg tamoxifen citrate/kg; ENVIGO) ad libitum for 14 days until tissue harvest. To inhibit VCP activity in vivo, CB5083 (Selleckchem) was dissolved in 0.5% methylcellulose + 5% Tween 80 (vehicle, filter sterilized) as a stock of 20 mg/mL to deliver a dose of 50 mg/kg by oral gavage q.o.d.

### Morphometric analyses.

Alveolar morphometry was performed using ImageJ. Volume density of alveolar septa, mean linear intercept of the airspaces, the mean transsectional wall length, and the surface area density of the air spaces were quantified following the protocol described previously (42).

### Lung IHC, immunofluorescence, and biochemistry.

Postnatal lungs were inflation fixed in 4% paraformaldehyde, embedded in paraffin, and sectioned at 5 μm. Imaging was done as described previously (20). Information on primary antibodies, secondary antibodies, dyes, and labeling reagents is listed in Supplemental Tables 2 and 3. To collect whole lung homogenates, frozen lung lobes were homogenized in PBS supplemented with Protease Inhibitor Cocktail (Sigma-Aldrich). Bronchoalveolar lavage fluid (BALF) was collected as described (40).

### AT2 cell isolation.

AT2 cells were isolated from postnatal lungs by cell depletion as described (43). CD45^–^, CD16/32^–^, Ter119^–^, CD90^–^, and CD31^–^ populations were collected as purified AT2 cells.

### Western blots.

Isolated AT2 and iAT2 cells were lysed in CelLytic M lysis buffer supplemented with Protease Inhibitor Cocktail. Cell debris was removed by centrifugation. SDS-PAGE and chemiluminescence were performed as described previously (20).

### Proteomic sample preparation.

Mass spectrometry–based proteomic analyses were performed at Pacific Northwest National Laboratory on isolated AT2 cells as described previously (20). Proteins were extracted from mice AT2 samples using the MPLEx method (44). Briefly, the samples were placed on ice in a mixture of water-chloroform-methanol (3:8:4). After gently mixing the samples several times for 5 minutes, samples were vortexed for 1 minute and centrifuged at 13,523 xg for 10 minutes at 4°C. The protein layer, which forms at the interface between the organic and hydrophilic phases, was harvested, rinsed with methanol, and air dried; bicinchoninic acid assay (BCA) was performed and 200 μg of proteins were subjected to trypsin digestion. Both copurified and mouse AT2–extracted proteins were solubilized in 50 mM NH_4_HCO_3_, 8 M urea, and DTT was added to reach 5 mM. Samples were incubated 30 minutes at 37°C with shaking at 800 rpm, iodoacetamide was added to reach a final concentration of 400 mM followed by a 60 minute incubation at 37 °C with shaking at 800 rpm. After a 10-fold dilution in 50 mM NH_4_HCO_3_, samples were digested overnight with trypsin (1:50 trypsin:protein ratio) and 1 mM CaCl_2_. Peptides from copurified proteins were desalted using a C18 ultramicrospin columns (3–30 μg capacity; Nest Group) and peptides from AT2 samples were desalted with C18 solid phase extraction (Strata, 50 mg/mL, Phenomenex). Samples were concentrated then evaporated using a speedvac to a volume of 25 μL.

### Proteomic LC-MS/MS run.

For the mouse AT2 cells, extracted peptides were diluted to 0.1 μg/μL while the copurifications, the concentration was lower than detectable by the BCA assay. 5 μL of samples were injected with a custom packed C18 column (70 cm × 75 μm i.d., Phenomenex Jupiter, 3 μm particle size, 300 Å pore size) coupled to a Waters NanoAquity UPLC system. Elution was carried out using the following gradient of water (solvent A) and acetonitrile (solvent B) both containing 0.1% formic acid: 1%–8% B for 2 minutes, 8%–12% B for 18 minutes, 12%–30% B for 55 minutes, 30%–45% B for 22 minutes, 45%–95% B for 3 minutes, hold for 5 minutes in 95% B, and 99%–100% B for 10 minutes. The murine AT2 peptides were analyzed on a QE-HFX Orbitrap instrument (Thermo Fisher Scientific), while the copurified peptides were analyzed using a Lumos Orbitrap (Thermo Fisher Scientific). Eluting peptides were analyzed online by nanoelectrospray ionization and MS1 scans were collected over 300–1800 *m/z* at a resolution of 60,000 at 400 *m/z*. The MS2 scans were collected in data-dependent acquisition with 2 second cycles and high-energy collision induced dissociation (HCD) fragmentation. For the murine AT2 peptides, 0.7 *m/z* isolation width, 35% normalized collision energy, and 45,000 resolution was used. For the copurified peptides, 2.0 *m/z* isolation width; 30% normalized collision energy, AND 7,500 resolution at 400 *m/z* before being dynamically excluded for 30 seconds for the copurified peptides was used.

### Murine AT2 peptide data analysis.

Data were searched with MaxQuant software (v.1.6.0.16) (45) against the mouse reference proteome database from Uniprot Knowledge Base (accessed March 18, 2021). Parameters were set as default unless otherwise specified. Peptides were required to be cleaved by trypsin at least at 1 termini, and 2 undigested sites were allowed per peptide. The following variable modifications were set as variable modifications: N-terminal acetylation (+42.0105), and methionine oxidation (+15.9949). Carbamidomethylation of cysteine residues was set as a fixed modification. Quantification was performed using the MaxQuant Label Free Quantification (MaxLFQ) method (PMID: 24942700). Data Analysis was carried using the RomicsProcessor package (v1.0, https://doi.org/10.5281/zenodo.3386526). Protein group LFQ intensities were log2 transformed, and the median was normalized within each sample ANOVA and 2-tailed Student *t* test. Missing Values were imputed using a downshifted normal distribution (46). Student *t* tests of normalized peptide intensities were used to determine protein or protein groups significantly altered in the various comparisons (*P* < 0.05). Significantly altered proteins groups were clustered and visualized in a *z* score normalized heatmap using pheatmap. Protein groups were converted to official gene symbols for further analyses. Functional enrichment analyses using Toppfun was performed on specific protein patterns. Significant hits poignant to this project were represented in a bar graph of –log_10_ (*P* value).

### Affinity copurified peptide data analysis.

Data were searched with MaxQuant software (v.1.5.5.1) (45) against the mouse reference proteome databased from Uniprot Knowledge Base (downloaded August 14, 2018). Peptides were required to be cleaved by trypsin in both termini, but 2 undigested sites were allowed per peptide. Modification parameters were set identically as for the murine AT2 searches. Quantification was performed using the intensity-based absolute quantification (iBAQ) method (47). Missing values were imputed with half the minimum value of the lowest iBAQ score in the quantification data matrix. Data were log_2_ transformed and median normalized within each sample. Student *t* tests of normalized peptide intensities were used to determine protein or protein groups significantly altered in the various comparisons (*P* < 0.05). Significantly altered protein groups were clustered and visualized in a *z* score–normalized heatmap using pheatmap. Protein groups were converted to official gene symbols for further analyses. Functional enrichment analyses using Toppfun was performed on specific protein patterns. Significant hits poignant to this project were represented in a bar graph of –log_10_ (*P* value). To identify potential EMC3 protein-protein interaction (PPI) partners, we utilized the R based AP-MS analyses package apmsWAPP, and specifically the sub package TSPM, which utilizes a 2-stage-Poisson model to determine significant PPIs (48). Spectral counts were normalized using the “DESeq” designation and proteins were filtered using “overallVar” with a var.cutoff = 0.1. Proteins with a *P* < 0.05 were deemed potential EMC3 partners and were visualized in a *z* score–normalized heatmap of the TSPM-normalized spectral counts. Functional enrichment analyses of potential EMC3 PPI partners were determined using Toppgene’s Toppfun (49). Significant hits poignant to this project were represented in a bar graph of the –log_10_ (*P* value).

### Human lung tissues.

Lung tissues from deidentified normal donors were provided by G. Pryhuber from the BRINDL program at the University of Rochester, Rochester, New York, USA. Lung tissues from deidentified patients with SFTPC^I73T^ mutation were provided by William A. Gower at the University of North Carolina School of Medicine, Chapel Hill, North Carolina, USA. The donors included donor no. 1, 8 years old; no. 2, 25 years old, and patients included patient no. 1, female, lung biopsy collected around 1 year old for diagnosis, early in ILD course; and no. 2, male, lung explant collected at lung transplant, with end stage ILD.

### RNA analyses.

RNA isolation, reverse transcription, and qPCR were performed as described previously (20) and using primers listed in Supplemental Table 4. *Sftpc* RNA was detected using Taqman probe Mm00488144_m1 and normalized to the levels of 18S RNA (TaqMan probe 4352930E, Applied Biosystems).

### Measurement of subcellular distribution of proSP-C.

Confocal immunofluorescence images were taken on lung sections stained for proSP-C, cell-surface marker WGA and endosome marker EEA1. Mean fluorescence intensity of total proSP-C and proSP-C colocalized with WGA or EEA1 was measured using ImageJ. Subcellular distribution of proSP-C was quantified as ratio of proSP-C colocalized with WGA or EEA1 to total proSP-C.

### iAT2 cell culture.

iAT2 cells carrying the *SFTPC^I73T^* mutation (clone SPC2-ST-C11) and their gene-corrected counterparts (clone SPC2-ST-B2) were generated and cultured as described (14).

### Statistics.

Statistical analysis was done on Prism 8. Values are expressed as the mean ± SEM. Statistical analysis was done on GraphPad Prism 8 (GraphPad Software). Significant differences were assessed by 2-tailed Student’s *t* test, 1-way ANOVA, or 2-way ANOVA. Data are expressed as the mean ± SEM. All graphs include means with error bars to show the distribution of the data. A *P* value of 0.05 or less was considered statistically significant.

### Study approval.

Mice were housed in pathogen-free facilities according to protocols approved by the IACUC of the Cincinnati Children’s Hospital Research Foundation and the Greater Bay Area Institute of Precision Medicine (Guangzhou, China). Human lung tissue/lung biopsies were provided by G. Pryhuber from the BRINDL program at the University of Rochester and William A. Gower at the University of North Carolina School of Medicine. Parent/guardian consent was obtained for use of excess clinical tissue under IRB protocol no. 2018-0852 of the Cincinnati Children’s Hospital Research Foundation.

### Data availability.

The mass spectrometry proteomics data on isolated AT2 cells and Myc-EMC3 copurified peptides have been deposited to the ProteomeXchange Consortium via the PRIDE (50) partner repository with the dataset identifiers PXD029962 and PXD017773 respectively. A Supporting Data Values file is provided in the supplemental materials.

## Author contributions

XT, WW, and YS performed all mouse studies and cell culture work. GC and ESN performed mass spectrometry and proteomic analyses. CLN performed the electron microscopy. JMS analyzed the mass spectrometry and proteomic data. KSA, EPM, and TEW developed and characterized the *Sftpc^I73T^* knock-in mouse model. DNK and KDA produced mutant and corrected iAT2 cells. JH and JDM performed and analyzed Seahorse assays. WAG collected and analyzed *SFTPC^I73T^* patient samples. XT, TEW, XL, and JAW interpreted the experiments. XT, TEW, and JAW designed the experiments and wrote the manuscript.

## Supplementary Material

Supplemental data

Unedited blot and gel images

Supporting data values

## Version 1 10/15/2024

In-Press Preview

## Version 2 12/02/2024

Electronic publication
