# Supplementary material for: EMC3 regulates trafficking and pulmonary toxicity of the SFTPCI73T mutation associated with interstitial lung disease
Source: J Clin Invest. 2024 Oct 15;134(23):e173861. doi: 10.1172/JCI173861 (PMC11601914; doi:10.1172/JCI173861)
Supplement: Supplemental data [file jci-134-173861-s136.pdf]

Supplemental Data

EMC3 regulates trafficking and pulmonary toxicity of the *SFTPC*^{I73T} mutation associated with interstitial lung disease

Authors: Xiaofang Tang[1,2*], Wei Wei[1], Yuqing Sun[1], Timothy E. Weaver[2], Ernesto S. Nakayasu[3], Geremy Clair[3], John M. Snowball[2], Cheng-Lun Na[2], Karen S. Apsley[2], Emily P. Martin[2], Darrell N. Kotton[4], Konstantinos-Dionysios Alysandratos[4], Jiuzhou Huo[5], Jeffrey D. Molkentin[5], William A. Gower[6], Xinhua Lin[1], Jeffrey A. Whitsett[2*]

Supplemental Methods

Supplemental Figures 1-11

Supplemental Tables 1-4

References

Supplemental Methods

Lung immunohistochemistry and immunofluorescence

Postnatal lungs were inflation fixed in 4% paraformaldehyde, embedded in paraffin, and sectioned at 5 µm. Imaging was done as described (1). Information on primary antibodies, secondary antibodies, dyes and labeling reagents used in this study is listed in Supplemental Table 2 and 3.

Hydroxyproline assay

The content of hydroxyproline in the lungs was analyzed using a hydroxyproline assay kit (Cat.# A030-2-1, Nanjing Jiancheng Bioengineering Institute). Briefly, for individual sample, a total of 30-100 mg of lung tissue was hydrolyzed, and pH of the supernatant was adjusted to 6.0-6.8. Blank controls, standards and samples for testing were prepared according to the manufacturer's instructions. Absorbance at 550 nm was then measured and the content of hydroxyproline in each sample was calculated using the formula below:

$$\text{Hydroxyproline Content}(\mu\text{g/mg tissue}) = \frac{A_{\text{sample}} - A_{\text{blank}}}{A_{\text{standard}} - A_{\text{blank}}} \times C_{\text{standard}} \times \frac{V}{W}$$

$C_{\text{standard}} = 5\mu\text{g/mL}$; $V = \text{Volume of Hydrolysate}$, 10mL ; $W = \text{Weight of Tissue}$

RNA analyses

RNA isolation, reverse transcription, and qPCR were performed as described (1). Primers used in the supplemental studies are listed in Supplemental Table 4. The transcript levels of *Acta2*, *Col1a1*, and *Col3a1* were normalized to that of β-actin. Levels of the spliced Xbp1 transcript (sXbp1) were normalized to that of the full-length Xbp1(uXbp1).

AT2 cell isolation

AT2 cells were isolated from postnatal lungs by cell depletion as described (2). $CD45^-$, $CD16/32^-$, $Ter119^-$, $CD90^-$, and $CD31^-$ populations were collected as purified AT2 cells.

Western blots

Isolated AT2, cultured MLE-15 cells and 293T cells were lysed in CelLytic M lysis buffer supplemented with Protease Inhibitor Cocktail. Cell debris was removed by centrifugation. SDS-PAGE and chemiluminescence were performed as described (1).

Electron microscopy

Postnatal lungs were inflation fixed with 2% paraformaldehyde (Electron Microscopy Sciences, EMS), 2% glutaraldehyde (EMS), 0.1% calcium chloride (Sigma-Aldrich), and 0.1 M sodium

cacodylate buffer, pH 7.3 (SCB; EMS) overnight at 4°C. Fixed lungs were cut into 1- to 2-mm blocks, postfixed with 1% osmium tetroxide (EMS) and 1.5% potassium ferrocyanide (Sigma-Aldrich) in 0.1 M SCB, pH 7.3, dehydrated in a graded series of alcohol, and infiltrated and embedded with EMbed 812 (EMS). Electron micrographs of lung sections were acquired using a Hitachi TEM H-7650 (Hitachi High Technologies America) with an AMT CCD camera (Advanced Microscopy Techniques).

Cell culture and transfection

MLE-15 cells were cultured as previously described (1). HEK293T cells (ATCC#: CRL-3216) were cultured in Dulbecco's modified Eagle medium (GIBCO, C11995500BT) supplemented with 10% fetal bovine serum (Hyclone, SH30406.05) and 1% penicillin–streptomycin (GIBCO, 15140122).

Cell transfection with plasmid and/or siRNA was done using the Lipofectamine 3000 reagent (Life Technologies) following the manufacturer's protocol. For Vcp silencing in MLE-15 cells, the following Silencer Select siRNAs (Thermo Fisher Scientific) were used for transfection: siControl (Cat.# 4457289), siVcp #1 (Cat.#s114337), and siVcp #2 (Cat.#s114338). For VCP silencing in HEK293T cells, the following siRNAs were used: siControl (Santa Cruz Biotechnology, sc-37007) and siVCP (Santa Cruz Biotechnology, sc-37187)

pIRES2-EGFP vector, pIRES2-EGFP-hSPC(1-187) and pIRES2-EGFP-hSPC(I73T)plasmids were previously described (3). Cells were harvested for immunofluorescence staining or Western blots 60 hours after transfection.

Supplemental figures

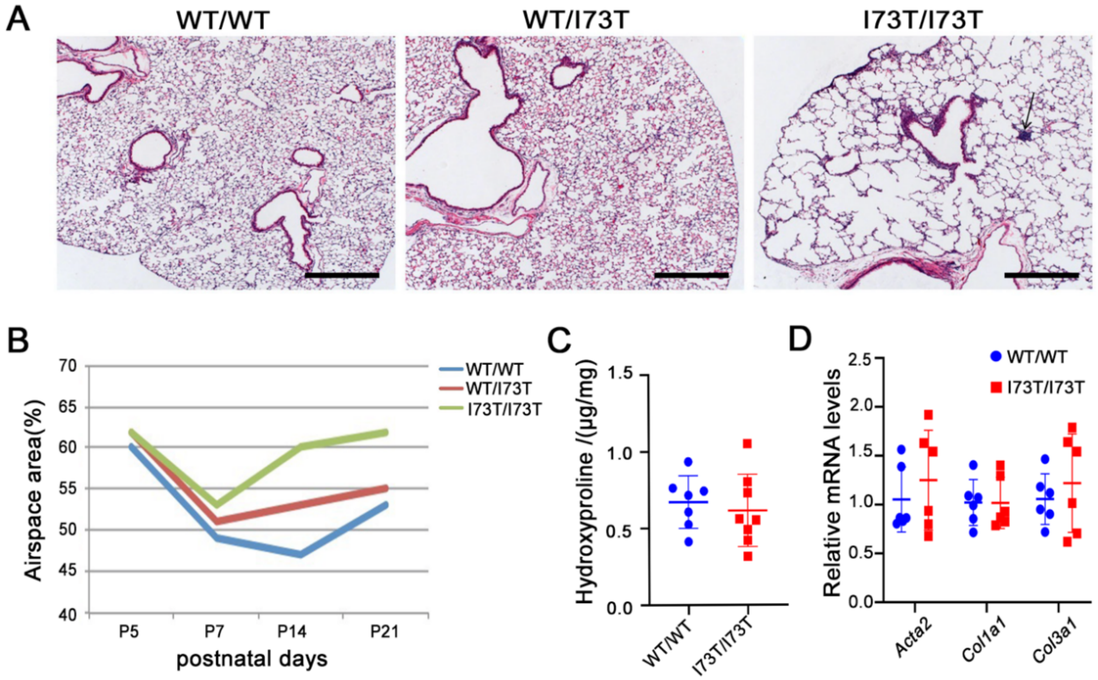

Supplemental Figure 1. Enlarged airspace in the postnatal lungs of *Sftpc*^{I73T} knock-in mice.
(A) Representative H&E-stained sections from 6-week *WT/WT*, *WT/I73T* and *I73T/I73T* mice. Arrows indicates lymphocytic aggregates. Scale bars, 400 μm.
(B) Quantification by ImageJ of airspace enlargement with age of *WT/WT*, *WT/I73T* and *I73T/I73T* mice as percentage of airspace area in the peripheral lung.
(C) Hydroxyproline content of *WT/WT* and *I73T/I73T* lungs at 6-8 weeks of age. No significant difference was observed between two groups. Mean ± SEM; n = 7 (*WT/WT*), n = 8 (*I73T/I73T*).
(D) mRNA levels of *Acta2*, *Col1a1*, and *Col3a1* in *WT/WT* and *I73T/I73T* lungs at 6-8 weeks of age. Levels of the transcripts were normalized to that of β-actin by qPCR. Comparable mRNA levels were detected between two groups. Mean ± SEM; n = 6/group.

.

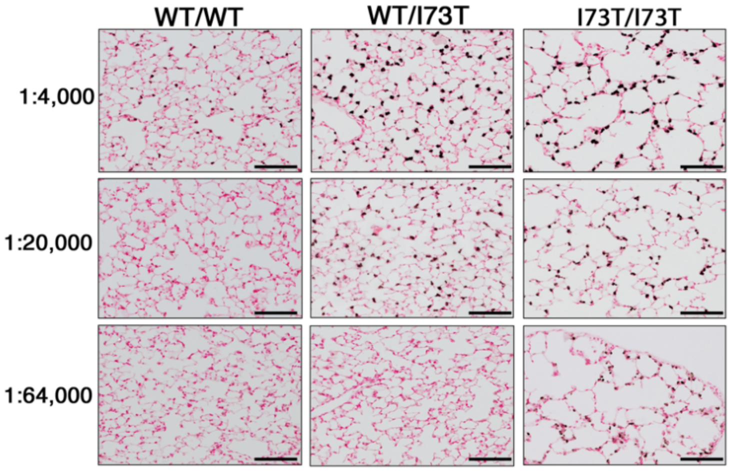

Supplemental Figure 2. Detection of proSP-C by a series of antibody dilutions.

SP-C(I73T) proprotein is readily detectable by proSP-C antibody staining at concentrations beyond dilution detecting SP-C(WT). Immunohistochemical staining (black signal) was performed on 6-week mouse lung sections. Scale bars, 100 µm.

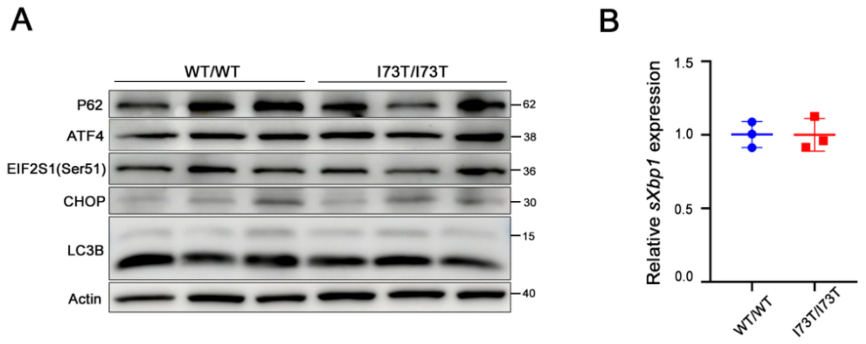

Supplemental Figure 3. ER stress and autophagy were unaltered in the AT2 cells of *Sftpc*^{I73T} knock-in mice.

(A) Western blot of lysates of AT2 cells isolated from *WT/WT* and *I73T/I73T* mice 6-8 weeks old detecting proteins involved in ER stress and autophagy. No major changes were detected between two groups.

(B) Xbp1 splicing in AT2 cells isolated from *WT/WT* and *I73T/I73T* mice 6-8 weeks old. Levels of the spliced Xbp1 transcript (sXbp1) were normalized to that of the full-length Xbp1(uXbp1) by qPCR. Mean ± SEM; n = 3/group.

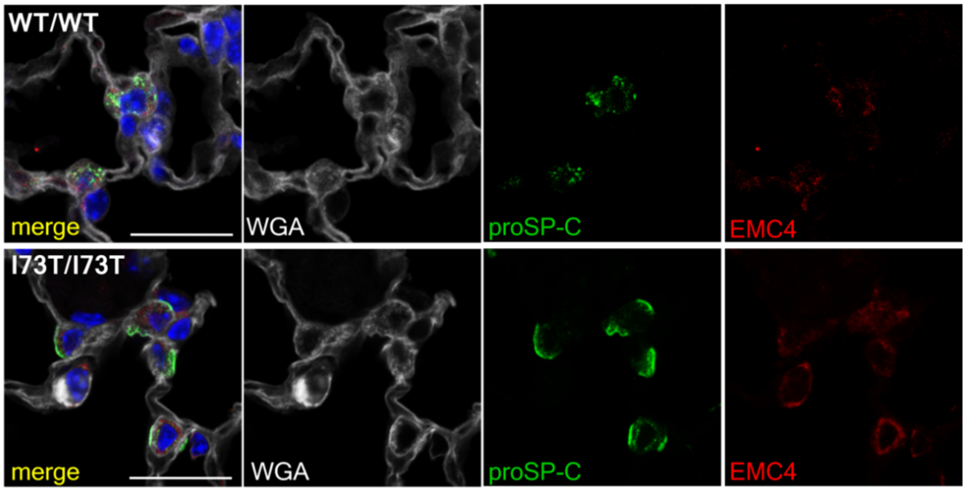

Supplemental Figure 4. Expression and distribution of EMC4 in AT2 cells of *Sftpc*^{I73T} knock-in mice.

Lung sections of 6-week-old *WT/WT* and *I73T/I73T* mice were stained for proSP-C (green), EMC4 (red), WGA (white) and DAPI (blue). Distinct staining patterns of EMC4 were detected in two genotypes. Scale bars, 20 µm.

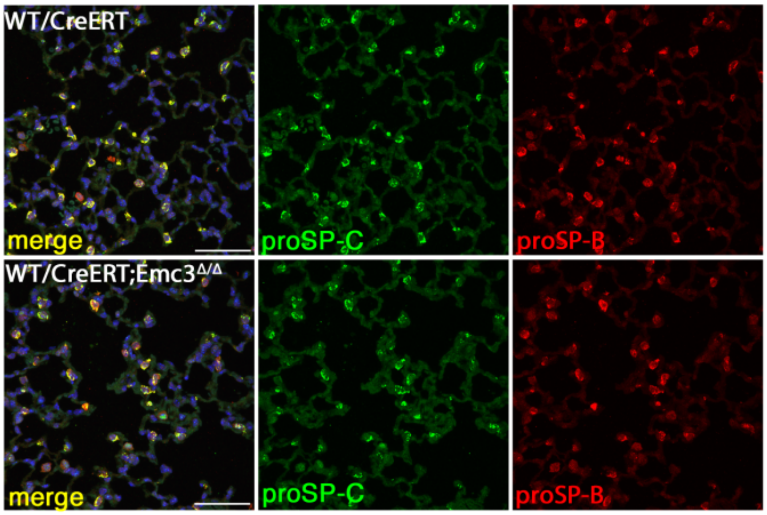

Supplemental Figure 5. Postnatal *Emc3* deletion in wild-type AT2 cells did not alter lung morphology or subcellular localization of surfactant proteins. AT2-specific deletion of *Emc3* was induced by injection of tamoxifen to neonatal mice on postnatal days 6, 7 and 8. Lung tissues from control (*WT/CreERT*) and *Emc3* deleted lungs (*WT/CreERT;Emc3*$^{\Delta/\Delta}$) were harvested on postnatal day 21 and stained for proSP-C (green), proSP-B (red) and DAPI (blue). Scale bars, 50 µm.

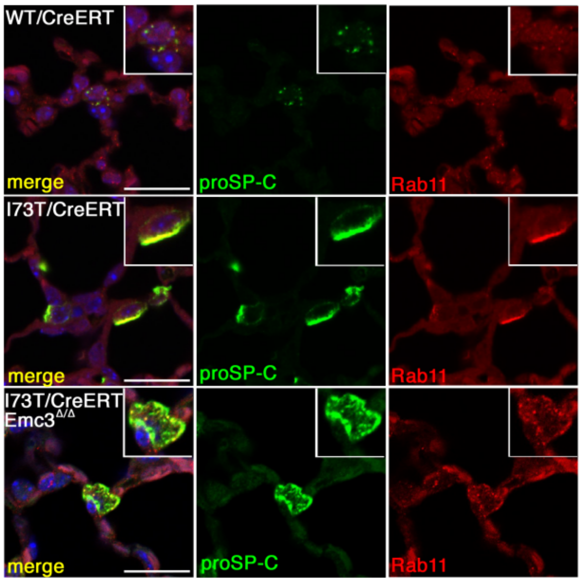

Supplemental Figure 6. *Emc3* deletion restored intracellular distribution of endosome marker Rab11. Control (*WT/CreERT*), *Sftpc*^{I73T} heterozygous (*I73T/CreERT*), and *I73T/CreERT;Emc3*$^{Δ/Δ}$ neonatal mice were treated with tamoxifen as in Figure 2A. Lung sections from P21 mice were stained for proSP-C (green), Rab11 (Red) and DAPI (blue). Compared to the diffuse intracellular staining of Rab11 in AT2 cells from *WT/CreERT* mice, Rab11 accumulated with SP-C(I73T) near the plasma membrane in *I73T/CreERT* mice. *Emc3* deletion restored the intracellular distribution of proSP-C(I73T) and Rab11. Scale bars, 20 μm.

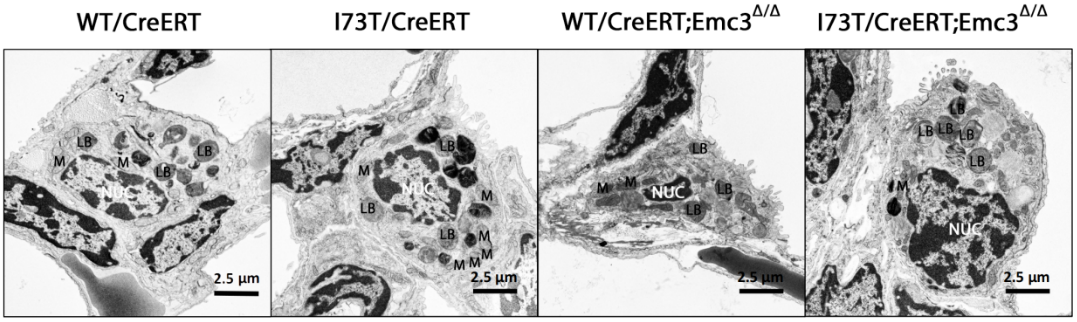

Supplemental Figure 7. Electron microscopic images of AT2 cells from tamoxifen treated mice at P21. Tamoxifen treatment was done on neonatal mice as in Figure 2A. Mice of four genotypes, control (*WT/CreERT*), *WT/CreERT;Emc3*$^{\Delta/\Delta}$, *Sftpc*^{I73T} heterozygote (*I73T/CreERT*), and *I73T/CreERT;Emc3*$^{\Delta/\Delta}$, were treated and subject to electron microscopic imaging. AT2 cells from *I73T/CreERT* lungs contained more mitochondria close to the cell surface, which was not observed with *I73T/CreERT;Emc3*$^{\Delta/\Delta}$ AT2 cells. Compared to the others, AT2 cells from *I73T/CreERT;Emc3*$^{\Delta/\Delta}$ lungs contained larger lamellar bodies. LB: lamellar body; M: mitochondrion; NUC: nucleus. Images are representative of n=3 mice of each genotype at P21.

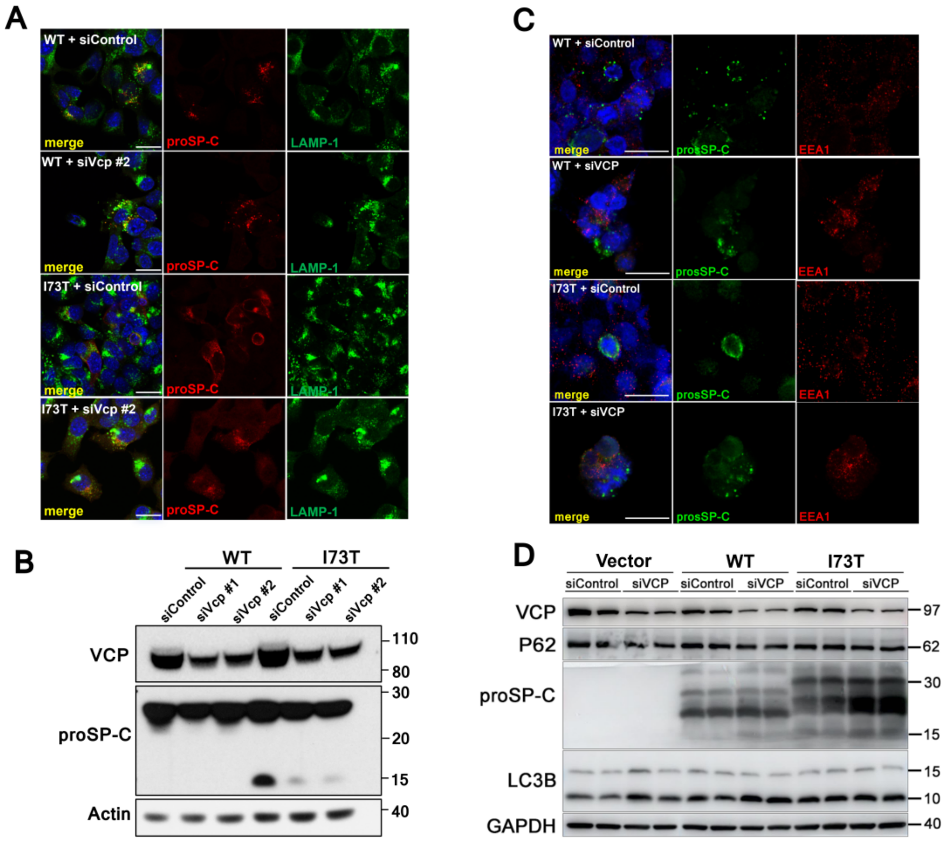

Supplemental Figure 8. RNAi-mediated knockdown of VCP altered the localization of proSP-C(I73T) in MLE-15 and HEK293T cells.

(A and B) MLE-15 cells were cotransfected with the same amount of plasmids encoding wild-type SP-C or SP-C(I73T) together with control siRNA (siControl) or two different Vcp siRNAs (siVcp #1 and siVcp #2). Cells were subject to immunofluorescence staining (A) or Western blotting analysis (B) 60 hours after transfection.

(C and D) HEK293T cells were cotransfected with the same amount of plasmids encoding empty vector, wild-type SP-C or SP-C(I73T) together with control siRNA (siControl) or VCP siRNA (siVCP). Cells were subject to immunofluorescence staining (C) or Western blotting analysis (D) 60 hours after transfection.

Scale bars, 20 µm.

Note that VCP knockdown did not affect the processing or trafficking of wild-typed SP-C in either cell lines. While different processing patterns of SP-C(I73T) were observed in two cell lines, VCP knockdown in both cases rendered the cell-surface proSP-C(I73T) to intracellular vesicles.

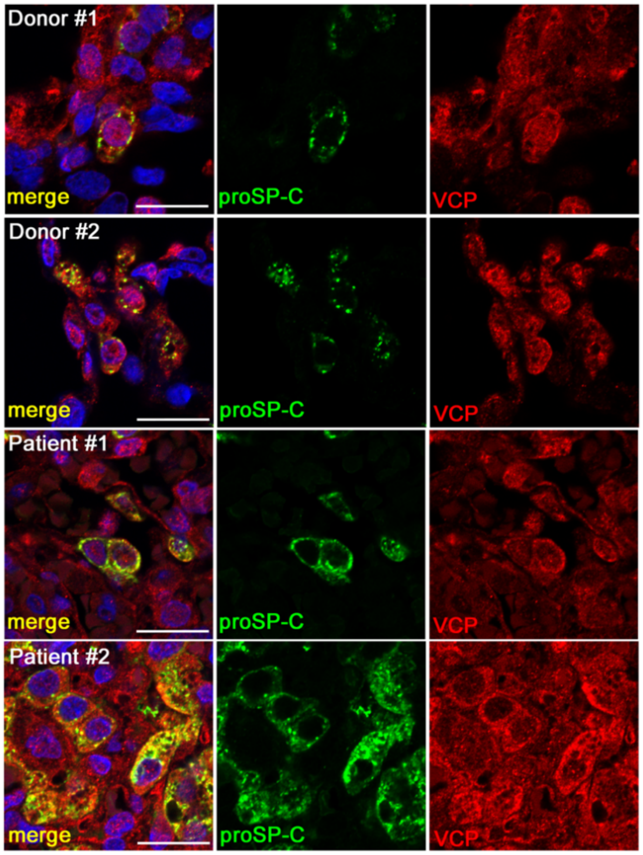

Supplemental Figure 9. Localization of SP-C(I73T) and VCP in patients with $\textbf{\textit{SFTPC}}^{\textbf{\textit{I73T}}}$-related interstitial lung diseases.

Lung sections from normal donors and patients with $\textit{SFTPC}^{\textit{I73T}}$-associated interstitial lung disease were stained for proSP-C (green), VCP (red) and DAPI (blue). With more proSP-C on the cell surface of patients' AT2 cells, VCP is readily detected around the cell surface in comparison to its widespread distribution pattern in control AT2 cells.

Scale bars, 20 µm.

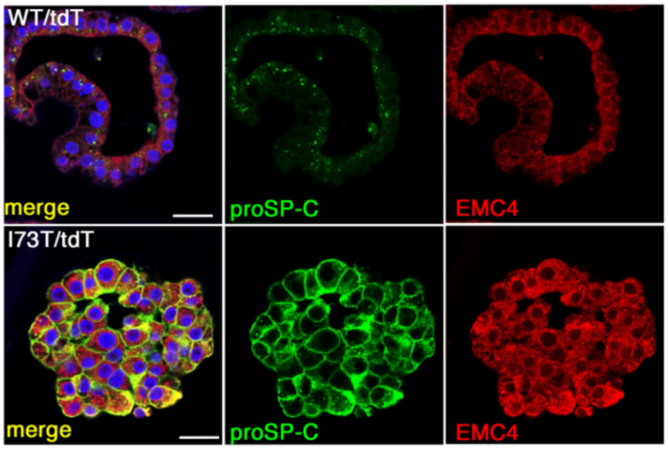

Supplemental Figure 10. Localization of proSP-C and EMC4 in AT2 cells differentiated from *SFTPC*^{I73T} patient-specific iPSCs. *SFTPC*^{I73T} patient-specific iPSCs and its gene-corrected wildtype counterpart were differentiated into I73T/tdT and WT/tdT AT2 (iAT2) cells respectively. iAT2 cell cultures were stained for proSP-C (green), EMC4 (red) and DAPI (blue), demonstrating the distinct structures of the alveolospheres and abnormal localization of proSP-C and EMC4 caused by *SFTPC*^{I73T} mutation. Scale bars, 20 µm.

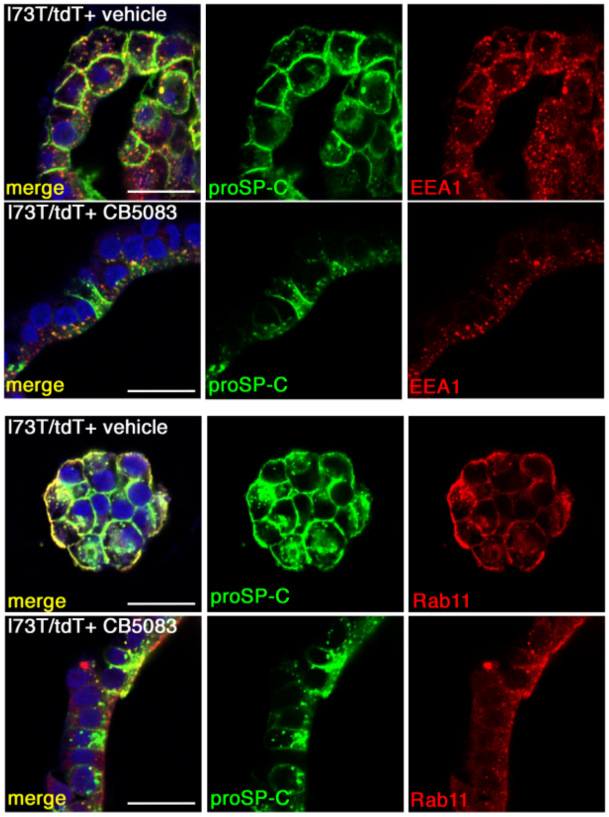

Supplemental Figure 11. CB5083 altered the localization of proSP-C(I73T) and endosomal markers EEA1 and Rab11 in AT2 cells differentiated from *SFTPC*^{I73T} patient-specific iPSCs. *I73T/tdT* iAT2 cells differentiated from *SFTPC*^{I73T} patient-specific iPSCs were treated as in Figure 8C and stained for proSP-C (green), EEA1 or Rab11 (red) and DAPI (blue). CB5083 altered the morphology of the cell culture and blocked peri-plasma membrane staining of proSP-C(I73T), EEA1 and Rab11. Scale bars, 20 µm.

Supplemental tables

Name	p-value	Hit Count in Query List	Hit Count in Genome	Hit in Query List
vesicle targeting, trans-Golgi to periciliary membrane compartment	1.27E-16	48	409	CETN2,CCDC114,TTC26,TUBB4B,WDR35,TTC30B,SCLT1,RSPH9,HSP90AA1,IFT22,POC1A,IFT74,TUBB,CFAP298,DNAH1,WDR19,PPP2R1A,ARL3,CFAP52,CEP135,CEP41,CEP131,HSPB11,SPAG17,IFT140,IFT172,SEPTIN9,CKAP5,DNAL1,AK7,DNAJB13,TEKT2,RSPH1,SYNE2,TEKT1,WDR90,DNAH5,DNAH8,TNPO1,UNC119B,DYNC2H1,ODF2,RFX2,RFX3,ACTR2,ATP6V0D1,RP1,PCM1
proteasomal protein catabolic process	4.05E-08	38	492	PARK7,RACK1,UBE2V2,SUMO1,UFD1,HSPA1A,HSPA1B,VCP,SGTA,DDI2,NPLOC4,ERLIN1,PPP2CB,UGGT2,SUMO2,PSMA1,PSMA2,PSMA3,PSMA4,UGGT1,PSMA6,PSMB5,PSMC1,PSMC2,PSMD3,PSMD8,PSMD9,PSMD11,DDI1,DDB1,NEDD4,SEC61B,NEDD4L,USP14,BCAP31,ERLIN2,BUB3,RPL11
vesicle fusion	7.17E-08	60	1003	LIN7C,ACTN1,OLA1,S100A11,TUBB4B,SERPINB6,MGST1,FER,TAGLN2,EEA1,ALDH3B1,ALDOA,HSPA1A,HSPA1B,HSPA8,VCP,CAP1,HSP90AA1,FN1,ANXA1,TUBB,IDH1,YWHAZ,PDXK,RHOA,SCRN2,FRK,RAB18,CCT8,DNAJC5,ATP6V0A1,CAND1,PSAP,MYO6,PSMA2,SPTBN2,PSMC2,PSMD3,PSMD11,GDI2,PGRMC1,LIN7A,LIN7B,PTPN6,CYB5R3,PYGB,GNAI2,WDR1,COPS5,GPI,PDCD6IP,HUWE1,COTL1,ACTR2,SCCPDH,PA2G4,CD47,ROCK1,LYN,STOM
cell cycle G2/M phase transition	1.65E-07	26	279	CETN2,TUBB4B,HSPA2,HSP90AA1,TUBB,PPP2R1A,CDK5RAP3,CEP135,CEP41,CEP131,PSMA1,PSMA2,PSMA3,PSMA4,PSMA6,PSMB5,CKAP5,PSMC1,PSMC2,PSMD3,PSMD8,PSMD9,PSMD11,NPM1,ODF2,PCM1
mitotic cell cycle	4.46E-07	61	1083	RPL26,CETN1,CETN2,PHB2,CLIP1,TUBB1,MET,TUBB4B,SLC9A3R1,FER,HSPA1A,HSPA1B,HSPA2,VCP,HSP90AA1,POC1A,ANP32B,ANXA1,TUBB,RHOA,RHOC,PPP2R1A,ARL3,CDK5RAP3,CEP135,CEP41,TNKS1BP1,CEP131,PSMA1,PSMA2,PSMA3,PSMA4,SPTBN1,PSMA6,PSMB5,CKAP5,PSMC1,PSMC2,PSMD3,PSMD8,PSMD9,PSMD11,DDB1,BANF1,PTPN6,EFHC1,SUGT1,GNAI1,RANBP1,PDCD6IP,TUBA1C,NUMA1,ODF2,REEP3,UNC119,PEBP1,SBDS,ROCK1,BUB3,PCM1,RPL17
proteasome	5.26E-07	11	45	PSMA1,PSMA2,PSMA3,PSMA4,PSMA6,PSMB5,PSMC1,PSMC2,PSMD3,PSMD8,PSMD11
regulation of ubiquitin-specific protease activity	4.79E-03	2	4	PARK7,VCP

Supplemental Table 1. Functional enrichment analysis of proteins generally repressed by *Emc3* deletion in Figure 4A using Toppfun. Methods used for the analysis are described in the main text. Proteins involved in each biological process and corresponding p-value are listed.

Antigen	Cat#	Supplier	IgG type	Dilution
EMC4	ab184544	Abcam	Rabbit IgG	1:100(IF) 1:100,000(WB)
BiP	G9043	Sigma Aldrich	Rabbit IgG	1:10,000(WB)
proSP-B	WRAB-55522	Seven Hills Bioreagents	Rabbit IgG	1:200(IF) 1:15,000(WB
mSP-B	WRAB-48604	Seven Hills Bioreagents	Rabbit IgG	1:200(IF) 1:15,000(WB
mSP-B	generated in the Whitsett laboratory		Guinea pig IgG	1:200(IF)
proSP-C	WRAB-9337	Seven Hills Bioreagents	Rabbit IgG	1:500(IF) 1:15,000(WB)
proSP-C	generated in the Whitsett laboratory		Guinea pig IgG	1:500(IF)
mSP-C	WRAB-76694	Seven Hills Bioreagents	Rabbit IgG	1:200(IF) 1:15,000(WB)
ABCA3	WRAB-70565	Seven Hills Bioreagents	Rabbit IgG	1:200(IF)
Actin	LMAB-C4	Seven Hill Bioreagents	Mouse IgG1	1:10,000(WB)
LAMP1, mouse	1D4B	Developmental Studies Hybridoma Bank	Rat IgG2a	1:200(IF)
LAMP1, human	H4A3	Developmental Studies Hybridoma Bank	Mouse IgG1	1:200(IF)
EEA1	3288S	Cell Signaling Technology	Rabbit IgG	1:100(IF)
Rab11	5589S	Cell Signaling Technology	Rabbit IgG	1:100(IF)
TOM20	NBP1-81556	Novus Biologicals	Rabbit IgG	1:100(IF) 1:2,000(WB)
GAPDH	A300-641A	BETHYL	Rabbit IgG	1:2,000(WB)
Lysozyme	generated in the Weaver laboratory		Rabbit IgG	1:2,000(WB)
Actin	4970S	Cell Signaling Technology	Rabbit IgG	1:1,000(WB)
ABCA3	DF9245-100	Affinity Biosciences	Rabbit IgG	1:1,000(WB)
CHOP	15204-1-AP-50UL	Proteintech	Rabbit IgG	1:1,000(WB)
EIF2S1(Ser51)	28740-1-AP-50UL	Proteintech	Rabbit IgG	1:1,000(WB)
ATF4	ab184909	Abcam	Rabbit IgG	1:1,000(WB)
VCP	GTX101089	GeneTex	Rabbit IgG	1:1,000(WB)
p62	P0067-200UL	Sigma-Aldrich	Rabbit IgG	1:1,000(WB)
LC3A/B	12741S	Cell Signaling Technology	Rabbit IgG	1:1,000(WB)
LC3B	3868T	Cell Signaling Technology	Rabbit IgG	1:1,000(WB)
GAPDH	5174S	Cell Signaling Technology	Rabbit IgG	1:1,000(WB)

Supplemental Table 2. Primary antibodies used in this study

Abbreviation: IF, Immunofluorescence; WB, Western blot.

Item	Cat#	Supplier	Dilution
Wheat Germ Agglutinin (WGA), Alexa Fluor™ 488 conjugate	W11261	Invitrogen	1:1,000(IF)
DAPI	40727ES10	Yeasen	1:500(IF)
Alexa Fluor® 647 AffiniPure™ Donkey Anti-Mouse IgG (H+L)	715-605-150	Jackson ImmunoResearch Laboratories Inc.	1:400(IF)
Alexa Fluor® 647 AffiniPure™ Donkey Anti-Rat IgG (H+L)	712-605-150	Jackson ImmunoResearch Laboratories Inc.	1:400(IF)
Alexa Fluor® 488 AffiniPure™ Goat Anti-Rabbit IgG (H+L)	111-545-003	Jackson ImmunoResearch Laboratories Inc.	1:400(IF)
Alexa Fluor 594 AffiniPure Donkey Anti-Rabbit IgG (H+L)	34212ES60	Yeasen	1:200(IF)
Alexa Fluor 647 AffiniPure Donkey Anti-Rabbit IgG (H+L)	34213ES60	Yeasen	1:200(IF)
Alexa Fluor® 488 AffiniPure™ Goat Anti-Guinea Pig IgG (H+L)	106-545-003	Jackson ImmunoResearch Laboratories Inc.	1:400(IF)
Cy™3 AffiniPure™ Donkey Anti-Guinea Pig IgG (H+L)	706-165-148	Jackson ImmunoResearch Laboratories Inc.	1:200(IF)
Cy™5 AffiniPure™ Donkey Anti-Guinea Pig IgG (H+L)	706-175-148	Jackson ImmunoResearch Laboratories Inc.	1:400(IF)
HRP-conjugated Goat Anti-Rabbit IgG(H+L)	LF102	EpiZyme	1:1,000(WB)
HRP-conjugated Goat Anti-Mouse IgG(H+L)	LF101	EpiZyme	1:1,000(WB)
Goat anti-Rabbit IgG Antibody (H+L), Biotinylated	BA-1000-1.5	Vector Laboratories	1:200(IHC)

Supplemental Table 3. Secondary antibodies, dyes and labeling reagents used in this study

Abbreviation: IF, Immunofluorescence; WB, Western blot; IHC, immunohistochemistry.

Name	Sequence (5’- 3’)
β-actin	F: CGTTGACATCCGTAAAGACC
	R: CTAGGAGCCAGAGCAGTAATC
Acta2	F: GTCCCAGACATCAGGGAGTAA
	R: TCGGATACTTCAGCGTCAGGA
Col1a1	F: GCTCCTCTTAGGGGCCACT
	R: CCACGTCTCACCATTGGGG
Col3a1	F: ATATGCCCACAGCCTTCTACAC
	R: ACCAGTTGGACATGATTCACAG
uXbp1	F: CAGACTACGTGCACCTCTGC
	R: CAGGGTCCAACTTGTCCAGAAT
sXbp1	F: GCTGAGTCCGCAGCAGGT
	R: CAGGGTCCAACTTGTCCAGAAT

Supplemental Table 4. Primers used for qPCR in Supplemental Figures 1D and 3B.

References

1. Tang X, Snowball JM, Xu Y, Na CL, Weaver TE, Clair G, et al. EMC3 coordinates surfactant protein and lipid homeostasis required for respiration. *J Clin Invest.* 2017;127(12):4314-25.
2. Rindler TN, Stockman CA, Filuta AL, Brown KM, Snowball JM, Zhou W, et al. Alveolar injury and regeneration following deletion of ABCA3. *JCI Insight.* 2017;2(24).
3. Sitaraman S, Martin EP, Na CL, Zhao S, Green J, Deshmukh H, et al. Surfactant protein C mutation links postnatal type 2 cell dysfunction to adult disease. *JCI Insight.* 2021;6(14).
