# Supplementary material for: EMC3 regulates trafficking and pulmonary toxicity of the SFTPCI73T mutation associated with interstitial lung disease
Source: J Clin Invest. 2024 Oct 15;134(23):e173861. doi: 10.1172/JCI173861 (PMC11601914; doi:10.1172/JCI173861)

Full unedited gels

Figure 1

Figure 1F

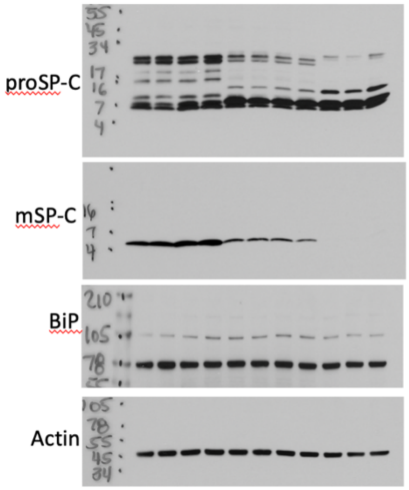

Figure 1G

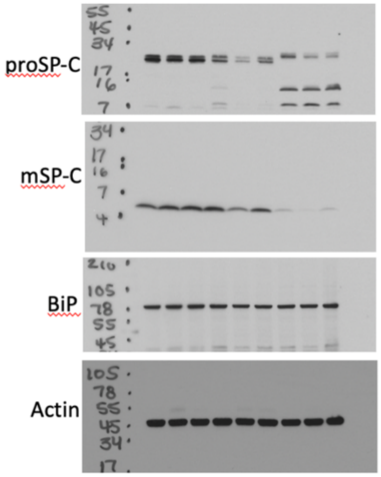

Figure 1H

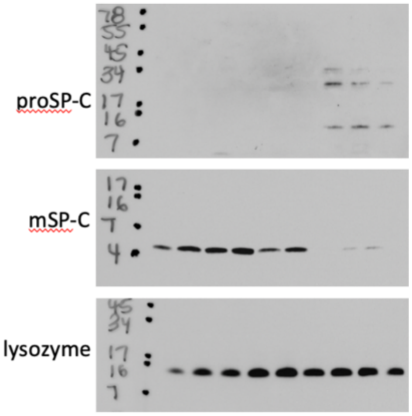

Figure 2

Figure 2C

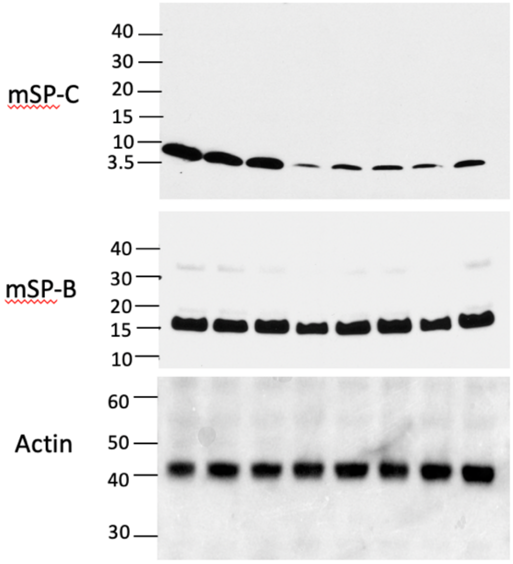

Figure 2E

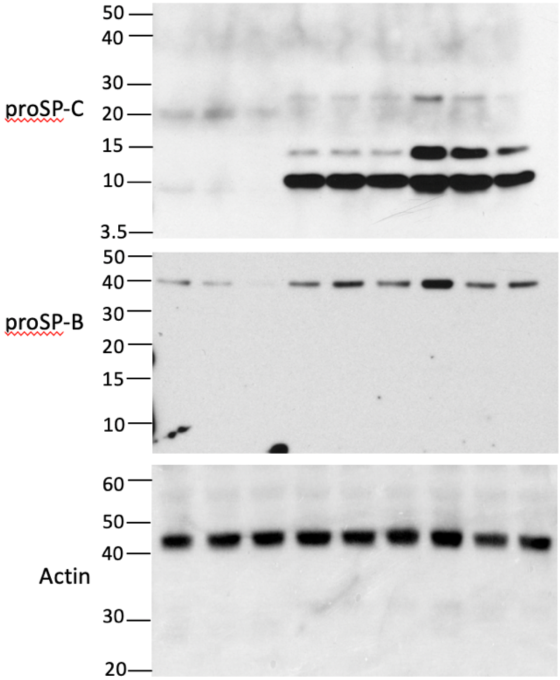

Figure 2

Figure 2G

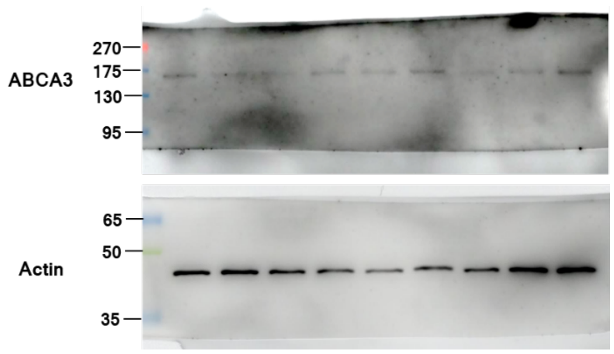

Figure 6

Figure 6G

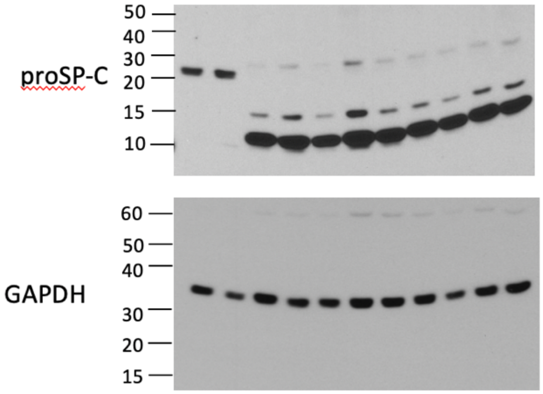

Figure 8

Figure 8B

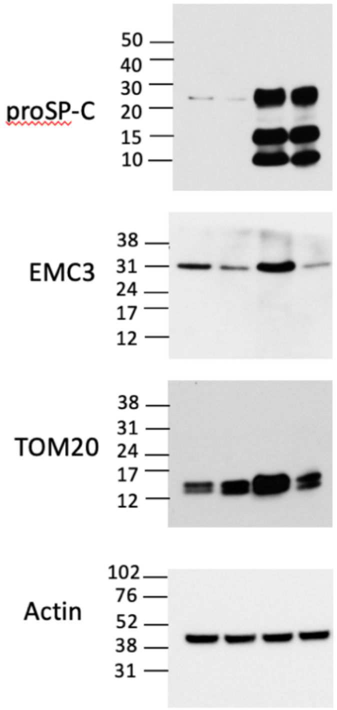

Figure 8D

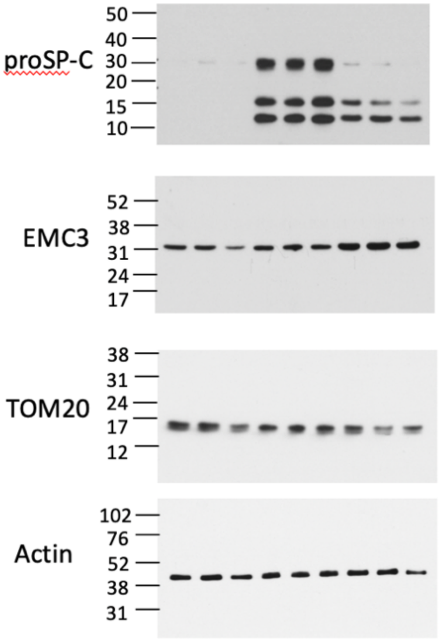

Figure S3

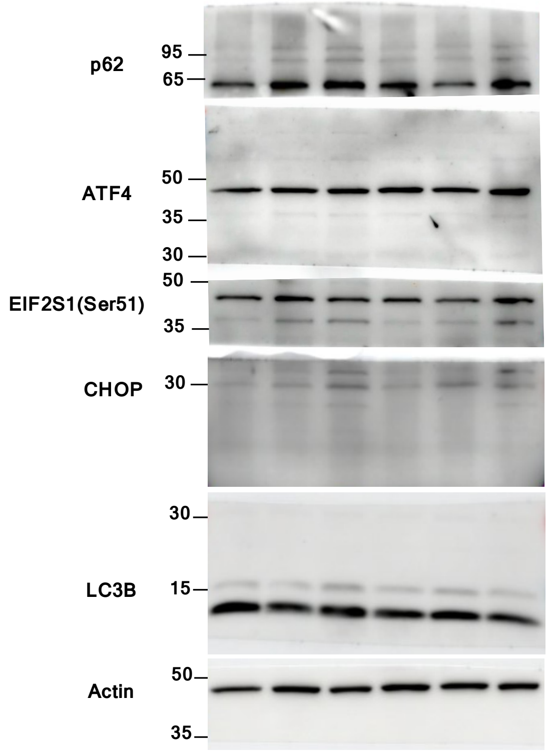

Figure S7B

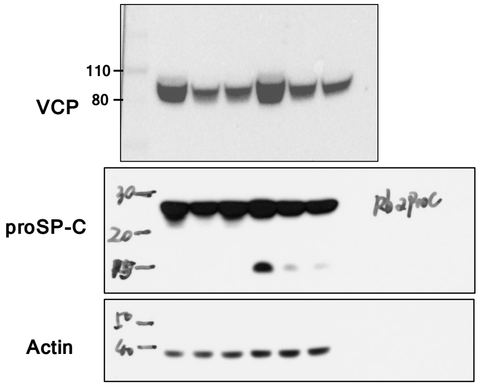

Figure S7D

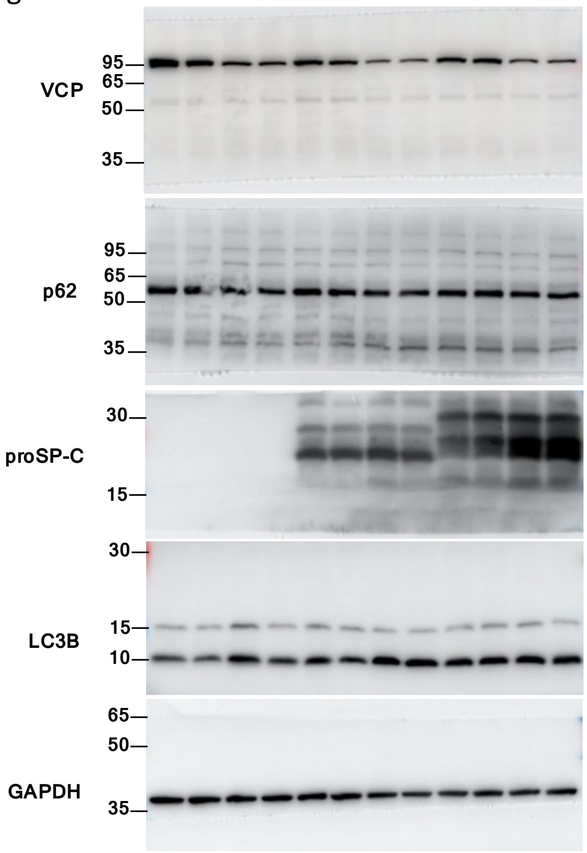

Supplement: Unedited blot and gel images [file jci-134-173861-s137.pdf]
